# Computational Simulations of Metal–Organic Frameworks to Enhance Adsorption Applications

**DOI:** 10.1002/adma.202405532

**Published:** 2024-07-27

**Authors:** Hilal Daglar, Hasan Can Gulbalkan, Gokhan Onder Aksu, Seda Keskin

**Affiliations:** ^1^ Department of Chemical and Biological Engineering Koç University Rumelifeneri Yolu, Sariyer Istanbul 34450 Turkey

**Keywords:** gas adsorption, metal–organic framework, molecular simulation

## Abstract

Metal–organic frameworks (MOFs), renowned for their exceptional porosity and crystalline structure, stand at the forefront of gas adsorption and separation applications. Shortly after their discovery through experimental synthesis, computational simulations quickly become an important method in broadening the use of MOFs by offering deep insights into their structural, functional, and performance properties. This review specifically addresses the pivotal role of molecular simulations in enlarging the molecular understanding of MOFs and enhancing their applications, particularly for gas adsorption. After reviewing the historical development and implementation of molecular simulation methods in the field of MOFs, high‐throughput computational screening (HTCS) studies used to unlock the potential of MOFs in CO_2_ capture, CH_4_ storage, H_2_ storage, and water harvesting are visited and recent advancements in these adsorption applications are highlighted. The transformative impact of integrating artificial intelligence with HTCS on the prediction of MOFs’ performance and directing the experimental efforts on promising materials is addressed. An outlook on current opportunities and challenges in the field to accelerate the adsorption applications of MOFs is finally provided.

## Graphical abstract

This review highlights the significance of molecular simulations in expanding the understanding of metal–organic frameworks (MOFs) and improving their gas adsorption applications. The historical development and implementation of molecular simulations in the MOF field are given, high‐throughput computational screening studies used to unlock the potential of MOFs in CO_2_ capture, CH_4_ storage, H_2_ storage, and water harvesting are discussed.

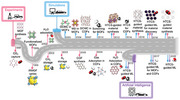

## 1 Introduction

Metal–organic frameworks (MOFs) are a class of crystalline materials consisting of metal ions/clusters coordinated to organic ligands via self‐assembly to form 1D, 2D, or 3D structures. MOFs are known for their highly porous nature and record‐breaking surface areas. Their internal surface areas reach up to ≈14 600 m^2^ g^−1^,^[^ ^1^ ^]^ surpassing those of traditional porous materials such as activated carbon (up to 2000 m^2^ g^−1^) and zeolites (up to ≈1500 m^2^ g^−1^). MOFs have a large variety of pore shapes and sizes ranging from 2 to 98 Å,^[^ ^2^ ^]^ and possess low densities, down to 0.124 g cm^−3^.^[^ ^3^ ^]^ All these remarkable structural properties, along with the high thermal, chemical, and mechanical stabilities observed in many MOFs, have made them very strong candidates for the storage of guest molecules in their pores, and ultimately MOFs have been widely studied for a variety of applications, including but not limited to gas adsorption, gas separation, drug storage, catalysis, and chemical sensing.^[^ ^4^ ^]^

MOFs have been synthesized using different types of methods such as solvothermal,^[^ ^5^ ^]^ microwave,^[^ ^6^ ^]^ electrochemical,^[^ ^7^ ^]^ mechanochemical,^[^ ^8^ ^]^ and sonochemical^[^ ^9^ ^]^ techniques. Their chemical and physical properties can be tuned for a target application during the synthesis specifically by combining different metal ions and organic ligands, and they can be functionalized after the synthesis to improve their performances. This chemical tunability, perhaps the most significant feature that distinguishes MOFs from traditional porous materials, has led to a very large number of materials having enormous structural diversity and functional versatility. We are currently aware of 125 383 different types of synthesized MOFs (obtained from the Cambridge Structural Database, CSD, using the Conquest^[^ ^10^ ^]^ tool on April 5, 2024) and millions of computer‐generated (hypothetical) MOFs. All these MOF materials offer great potential for energy, environment, and biomedical technologies. Many of these MOFs have been widely studied by researchers across the world using various experimental and computational techniques to identify the most useful materials for the desired storage applications.

## 2 Background of the MOF Field

**Figure 1:**
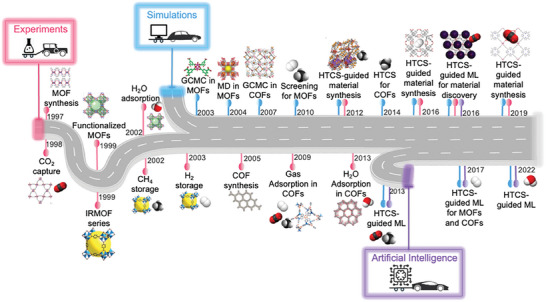
Milestones in the MOF literature, which started with experimental synthesis studies, and then evolved into a rich field combining experiments, theory, and data science to enable the design and discovery of new porous materials that will change our world, especially for clean energy, clean air, and clean water applications.

We illustrated how MOF research has evolved from past to present combining experiments, molecular simulations, and, very recently, artificial intelligence (AI) in **Figure** **1** and highlighted the milestones in the field. Historically, the concept of MOFs began to emerge when researchers explored the ways to synthesize porous materials by linking metal ions with organic molecules. Research beginning in the 1960s with the synthesis of a coordination polymer, bis(adiponitrilo) copper(I) nitrate, continued in the 1990s and significant efforts were invested in synthesizing materials that could be classified as coordination networks or coordination polymers,^[^ ^11^ ^]^ which are defined as “solid materials formed by an extended network of metal ions coordinated to multidentate organic molecules.”^[^ ^12^ ^]^ The definition of a coordination polymer did not differentiate between crystalline and amorphous materials, porous and nonporous structures, or robust and unstable solids. The term “MOF” was first used to present the hydrothermal synthesis of a crystalline material having large rectangular channels by Yaghi and Li in 1995.^[^ ^13^ ^]^ MOFs are defined as “a subclass of coordination polymers and they are crystalline and porous compounds involving strong metal–ligand interactions.”^[^ ^14^ ^]^

At that time, the main challenge was to achieve permanent porosity and to prevent the collapse of the crystalline structure in the absence of solvent molecules. In 1999, Yaghi's team made a groundbreaking contribution by synthesizing MOF‐5 (also known as isoreticular MOF‐1, IRMOF‐1) composed of zinc oxide (Zn_4_O) clusters and 1,4‐benzenedicarboxylic acid (BDC) linkers at the pcu topology and showed that this crystalline structure remains stable even when desolvated and heated up to 300 °C.^[^ ^15^ ^]^ The BDC linker enabled the formation of a 3D framework with a higher surface area (3800 m^2^ g^−1^) and pore volume (1.36 cm^3^ g^−1^) compared to most zeolites. In 2002, the same group constructed isoreticular MOFs (IRMOFs), having distinct physical and chemical characteristics such as pore sizes varying from 3.8 to 28.8 Å by using Zn_4_O clusters and different organic linkers.^[^ ^16^ ^]^ This approach, namely, “reticular chemistry,” encompassed the strategic assembly of rigid molecular building blocks into ordered structures, which are stabilized by the presence of strong bonds.^[^ ^17^ ^]^ It is a simple yet potentially universal design strategy and is now being explored for the synthesis of MOF materials, representing a significant advancement in the field of chemistry and material science.

First studies on the MOF synthesis directed the research community to investigate the potential of these new materials for gas storage applications. In 1997, Kitagawa and co‐workers synthesized a Co‐based MOF with bipyridine linkers and demonstrated that its CH_4_, N_2_, and O_2_ uptakes were comparable to conventional zeolites.^[^ ^18^ ^]^ Yaghi's group measured CO_2_ and N_2_ adsorption isotherms of Zn(BDC) MOF and showed that gas adsorption and release processes are reversible, similar to zeolites, without any unexpected retention of gases.^[^ ^19^ ^]^ In 1999, MOF‐5 was tested for Ar, N_2_, and organic vapor (CH_2_Cl_2_, CHCl_3_, CCl_4_, C_6_H_6_, C_6_H_12_) adsorption and reversible isotherms were reported for all guest molecules.^[^ ^15^ ^]^ IRMOF materials were tested for CH_4_ storage^[^ ^16^ ^]^ and H_2_ storage.^[^ ^20^ ^]^ IRMOF‐6 was reported to have high CH_4_ uptake (155 cm^3^ STP cm^−3^) at 298 K and 36 atm, exceeding zeolite 5A (87 cm^3^ STP cm^−3^) whereas IRMOF‐6 and IRMOF‐8 exhibited two (1 wt%) and four times (2 wt%) higher H_2_ uptake capacity than MOF‐5 (0.5 wt%), respectively, at 298 K, 10 bar. In 1999, a new MOF, Cu‐BTC, having benzene‐1,3,5‐tricarboxylate (BTC) linker was synthesized^[^ ^21^ ^]^ and tested for CO_2_, CH_4_, N_2_, and O_2_ adsorption in 2002.^[^ ^22^ ^]^ Cu‐BTC has then become one of the most extensively studied MOFs for gas adsorption thanks to its high surface area, high chemical stability, and easy synthesizability.^[^ ^23^ ^]^

As shown in Figure 1, covalent organic frameworks (COFs), COF‐1 and COF‐5, were first synthesized in 2005 by Yaghi and co‐workers.^[^ ^24^ ^]^ COFs, positioned as nonmetallic counterparts to MOFs, possess enhanced chemical stability thanks to their covalently bonded organic units within their crystalline structures.^[^ ^25^ ^]^ Their high surface areas and low densities make them promising candidates to be utilized for gas storage applications.^[^ ^26^ ^]^ COF‐5 was reported to have a higher surface area (1670 m^2^ g^−1^) and pore volume (1.07 cm^3^ g^−1^)^[^ ^27^ ^]^ compared to mesoporous ordered silica, MCM‐41 (680 m^2^ g^−1^ and 0.26 cm^3^ g^−1^,^[^ ^28^ ^]^ respectively). Initial experiments demonstrated that COF‐5 has H_2_ storage capacity of 3.4 wt% at 50 bar and 77 K.^[^ ^29^ ^]^ Subsequent studies on various COFs, including COF‐1, ‐6, ‐8, and ‐10 reported H_2_ uptakes of 1.5, 2.3, 3.5, and 4 wt%, respectively.^[^ ^30^ ^]^ COF‐102 and COF‐103 exhibit much higher H_2_ capacities, ∼7 wt%, at ≈40 bar and 77 K, surpassing the target of 5.5 wt% gravimetric capacity set by the U.S. Department of Energy (DOE) for H_2_ storage.^[^ ^30^ ^]^ As of today, there are ≈1250 different types of experimentally reported COFs,^[^ ^31^ ^]^ and hundreds of thousands of hypothetically designed COFs.^[^ ^32^ ^]^

From the synthesis of the first MOF in 1995, the number of synthesized materials has immensely grown and the crystal structures of MOFs have been deposited into the CSD.^[^ ^33^ ^]^ The number of synthesized MOFs climbed to thousands in the 2000s and increased exponentially to hundreds of thousands in the 2020s. In addition to experimentally synthesized MOFs available in the CSD, trillions of hypothetical MOFs (hMOFs) have been computationally generated (but not synthesized yet except a few) by iterating through potential combinations of building units, metals, and linkers, across various topologies. The first hMOF database^[^ ^34^ ^]^ consisting of 137 953 different types of structures was reported in 2012. These hMOFs were constructed by using the information of 102 building blocks, including 84 organic linkers, 5 metal nodes, and 13 functional groups, and 6 topologies that have been observed in the already synthesized MOFs. Numerous additional hMOFs offering a very wide range of chemical compositions have been then reported. The establishment of these large MOF material databases having a wide physical and chemical diversity has been useful for extracting the structure–performance relationships of MOFs and tuning their properties for the desired applications, which accelerates the identification of promising MOFs by reducing the time and resources required for new materials discovery.

Considering the existing and ever‐growing number of synthesized and hypothetical MOFs and COFs, it has become impractical to examine every single material, even for a single target application, using only experimental techniques. Molecular simulations are powerful, state‐of‐the‐art methods that can complement experimental efforts and, in certain cases, can replace them to provide atomic‐level information about the materials’ properties and to study the materials under challenging conditions with remarkable accuracy. Molecular simulations have been used to assess the properties of MOFs starting from the early 2000s, a few years after their experimental investigations, as shown in Figure 1. Early simulation studies focused on a limited number of MOF structures and adsorption of distinct types of gas molecules. In 2003, the first molecular simulation study using the grand canonical Monte Carlo (GCMC) technique examined Ar adsorption in one of the first synthesized MOFs, Cu‐BTC, between 10^−6^ and 1 atm, at 87.3 K.^[^ ^35^ ^]^ GCMC is a simulation method to mimic an adsorption experiment by keeping the chemical potential (*µ*), volume (*V*), and temperature (*T*) constant and allowing the number of gas molecules to fluctuate in the simulation box. The results of GCMC simulations for Ar adsorption in Cu‐BTC were mostly in good agreement with those of experimental isotherms, and preferential adsorption sites of Ar in the pores of the MOF were identified, demonstrating the ability of molecular simulations to accurately describe the gas adsorption behavior.

The first molecular dynamics (MD) simulations of MOFs appeared in 2004 and computed Ar diffusion in Cu‐BTC.^[^ ^36^ ^]^ MD simulations assess the time evolution of the positions and velocities of molecules by integrating Newton's equation of motion and they have been widely used to compute the transport properties of gases in MOFs, such as the self‐diffusion coefficients of gases in the pores of MOFs.^[^ ^37^ ^]^ Ar diffusivities in Cu‐BTC were simulated up to 10^4^ bar at 298 K and found to be within the same order of magnitude as those of silica zeolites, ITQ‐3 and ITQ‐7. Snurr and co‐workers then computed the adsorption and diffusion of larger gas molecules including CH_4_, n‐pentane (C_5_H_12_), n‐hexane (C_6_H_14_), and n‐heptane (C_7_H_16_) in IRMOF‐1 up to 5 kPa, at 300 K.^[^ ^38^ ^]^ The self‐diffusivities of n‐alkanes in IRMOF‐1 were found to be similar to those in porous crystalline bipyridines. These studies pioneered the field by providing a molecular‐level understanding of gas adsorption and diffusion in MOFs and significantly accelerated the use of molecular simulations to advance their use in guest storage and transport applications. Prior to the advent of high‐throughput computational screening (HTCS) and AI‐based techniques for evaluating the adsorption properties of very large number of MOFs, there are several excellent reviews that cover studies utilizing various molecular simulations for different MOFs and gases.^[^ ^39^ ^]^

In 2010s, HTCS approach utilizing molecular simulations was developed and then widely used to identify the most promising MOFs among thousands of candidate materials for several gas storage and separation applications.^[^ ^40^ ^]^ As the number of MOFs and their potential applications keep increasing, the HTCS approach has been an indispensable tool to find out the optimal MOF materials with desired properties and performances for target applications, specifically for CO_2_ capture,^[^ ^41^ ^]^ CH_4_ storage,^[^ ^41^ ^]^ H_2_ storage,^[^ ^42^ ^]^ and water harvesting.^[^ ^43^ ^]^ Today, these four applications have been the most mature, widely studied applications of MOFs and we will address the contribution of molecular simulations in these four fields in detail in the next chapter.

Today, the number of MOFs has reached very large numbers and even if a single novel MOF is synthesized, testing this material for many different applications is not practical. Studying every MOF for every possible application using molecular simulations is not efficient anymore due to the large computational time and requirement to perform molecular simulations. Data science, specifically AI, has emerged as a powerful approach to be integrated into the HTCS studies of MOFs to examine this large material spectrum for various applications and to efficiently analyze the resulting complex and extensive datasets for uncovering the specific molecular features that will guide the design of new materials with enhanced performance. This integration also facilitates the rapid identification of promising candidates by predicting materials’ properties and performance metrics, thereby reducing the reliance on time‐consuming and resource‐intensive experimental procedures. AI‐driven models can learn from the existing experimental and simulation datasets and identify patterns and multidimensional relationships that may not be immediately apparent through classical experiments and simulations, such as how gas uptakes in different temperatures and pressures correlate with the structural, chemical, and energetic properties of MOFs. Researchers recently utilized AI to automate the process of MOF synthesis conditions by extracting previously published experimental data, resulting in fast decisions for experimentalists about the synthesis of a target MOF.^[^ ^44^ ^]^ Today, self‐driving laboratories represent a groundbreaking integration of AI and robotics into the design and synthesis of materials, pushing the boundaries of automation and efficiency in experiments.^[^ ^45^ ^]^

Overall, we have witnessed that the MOF literature, which started with purely experimental synthesis studies, has evolved into a very rich scientific field combining experiments, theory, and data science to enable the design of novel materials that will change our world, especially for clean energy, clean air, and clean water applications.

## 3 Molecular Simulations of MOFs

### 3.1 Main Contributions of Molecular Simulations

Molecular simulations have significantly contributed to our understanding of MOFs and the development of application‐specific MOFs in the last two decades. We first addressed these contributions along with the representative examples below.

i) Providing atomic‐level information that is difficult to obtain from experiments

Molecular simulations offer atom‐based insights into the gas adsorption of MOFs under extreme conditions, such as high and/or low temperatures and/or pressures that cannot be easily reached by the experiments. For instance, Han et al.^[^ ^46^ ^]^ performed GCMC simulations at a wide range of temperatures from 77 to 300 K and up to 100 bar for a series of hexagonal IRMOFs to examine their H_2_ adsorption. The H_2_ adsorption in MOFs was found to mainly correlate with the heat of adsorption values at low pressure (1 bar), free volume at intermediate pressure (30 bar), and surface area at high pressure (100 bar) at 77 K. The introduction of Li doping into the MOFs resulted in a substantial increase in H_2_ uptake, particularly at 243 K. IRMOF‐2‐96‐Li exhibited a reversible H_2_ storage of 6.5 wt% at 243 K and 100 bar, meeting the DOE target (at least 6.0 wt% for temperatures above 243 K and pressures below 100 bar) at the time of publication.

Molecular simulations have been instrumental in elucidating the atom‐to‐atom interactions of MOFs with various guest molecules. For example, Gallo et al.^[^ ^47^ ^]^ studied H_2_ and CH_4_ storage in IRMOF‐11, MOF‐74, and MOF‐177 up to 80 bar at 298 K and showed a good agreement between experimental and simulated results. They showed that CH_4_‐framework and H_2_‐framework energetic interactions weaken with increasing pressure for MOF‐74 because of its smaller pore size and surface area, compared to IRMOF‐11 and MOF‐177, which force guest molecules into less energetically favorable sites. Diffusivities of gas molecules in MOFs’ pores, which are often challenging to measure experimentally, can be obtained using molecular simulations. For example, Skoulidas and Sholl performed MD simulations to compute the diffusivities of light gases (Ar, CH_4_, CO_2_, N_2_, and H_2_) in IRMOF‐1 at 298 K and showed that they are very similar to those in zeolite MFI.^[^ ^48^ ^]^ Keskin and Sholl combined GCMC and MD simulations to assess the gas flux through an IRMOF‐1 membrane and demonstrated that high CO_2_ fluxes could be achieved with MOF membranes.^[^ ^49^ ^]^ These studies revealed the potential of MOFs as adsorbents and membranes for separation applications.

Molecular simulations provide significant advantages in calculating the adsorption of gases in MOFs, especially for those difficult to measure experimentally. For instance, Li et al.^[^ ^50^ ^]^ used GCMC simulations to calculate the adsorption of H_2_S/CH_4_, H_2_S/CO_2_, SO_2_/CO_2_, and SO_2_/N_2_ mixtures on UiO‐66(Zr) and its seven different functionalized versions up to 20 bar and 298 K. The results indicated that the carboxylic acid functionalized MOF demonstrated significantly higher selectivity, making it effective in capturing toxic H_2_S and SO_2_. Another contribution of molecular simulations is their ability to calculate adsorption properties in multicomponent systems that are challenging to measure experimentally. For example, Peng et al.^[^ ^51^ ^]^ used GCMC simulations to determine the adsorption properties of CH_4_/CO_2_/H_2_S and N_2_/CO_2_/SO_2_ mixtures for 12 MOFs, zeolites, COFs, and carbon nanotubes (SWNTs) at 40 bar and 303 K. The results showed that the selectivities of MOFs for H_2_S and SO_2_ are quite high (up to 160 and 400) and comparable to those of zeolites and SWNTs.

ii) Guiding experimental efforts via unlocking new physical/chemical insights

The strategic focus of experimental endeavors in the field of MOFs is shifting toward identifying the most promising materials for a target application, and molecular simulations have an important role in designing novel MOFs that outperform already‐existing ones in gas adsorption applications. For instance, Karra and Walton studied the impact of pore size, heat of adsorption, and open metal sites (OMSs) of MOFs (IRMOF‐1, IRMOF‐3, Cu‐BTC, and Zn_2_[bdc]_2_[dabco]) on their adsorption performances and modeled their ligand functionalized versions to investigate their CO_2_ adsorption by GCMC simulations up to 25 bar at 298 K.^[^ ^52^ ^]^ Results revealed that small‐pored MOFs and large‐pored MOFs with open metal sites can exhibit high CO_2_ adsorption. They showed that amine‐functionalized IRMOF‐3 has enhanced CO_2_ adsorption capacities compared to IRMOF‐1 at lower pressures. This study has been pioneering in terms of guiding the synthesis of functionalized MOFs to achieve high CO_2_ adsorption. Snurr's group performed GCMC simulations for CH_4_ adsorption in the IRMOF family at 35 bar, 298 K.^[^ ^53^ ^]^ They first showed the good agreement between experimental and simulated adsorption isotherms for IRMOF‐1 and IRMOF‐6, and then generated hypothetical MOFs by changing the linkers of IRMOF‐1 to maximize the CH_4_ uptake. Results showed that CH_4_ uptake of the computer‐generated IRMOF‐993 structure (181 cm^3^ STP cm^−3^) exceeded the target value of 180 cm^3^ STP cm^−3^ defined by the DOE at the time of publication. Froudakis and co‐workers examined the H_2_ adsorption in MOFs by computationally modifying the organic linker of IRMOF‐10 with Mg^2+^ and found that the interaction energy of the modified linker is more than five times higher than that of the single aromatic linker.^[^ ^54^ ^]^ GCMC simulations were subsequently performed and a constant increase in the volumetric H_2_ uptake was demonstrated thanks to the stronger binding sites upon Mg^2+^ modification up to 100 bar, at 77 and 300 K.

MOFs exhibit remarkable structural versatility in response to various stimuli such as pressure, temperature, light, and guest molecules, making them dynamic and adaptable materials. The implications of these experimentally observed properties on the gas adsorption characteristics of MOFs have been extensively analyzed through molecular simulations. For instance, Wang et al.^[^ ^55^ ^]^ explored the C_3_H_8_/C_3_H_6_ separation in the flexible MOF, NJU‐Bai8 using GCMC simulations, revealing an Ideal Adsorbed Solution Theory (IAST) selectivity of 4.6 at 0.2 bar and 298 K. Similarly, Li et al.^[^ ^56^ ^]^ examined the flexible MOF, Cu(dhbc)_2_(4,4′‐bipy) for mixtures consisting of CH_4_, C_2_H_2_, C_2_H_4_, C_2_H_6_, C_3_H_4_, C_3_H_6_, and C_3_H_8_, determined the IAST selectivities at 273 and 298 K, and identified the highest selectivity for C_3_H_4_. Garcia‐Perez et al.^[^ ^57^ ^]^ used GCMC simulations to study CO_2_ and CH_4_ adsorption in the flexible MOF, NH_2_‐MIL‐53, finding good agreement with experimental results. Fairen‐Jimenez et al.^[^ ^58^ ^]^ evaluated the adsorption of CO_2_, CH_4_, C_2_H_6_, C_3_H_8_, and C_4_H_10_ in the flexible ZIF‐8 using GCMC simulations and obtained excellent agreement between simulated and experimental adsorption data. MOFs featuring OMSs present unique opportunities for enhancing the adsorption affinity toward specific adsorbates, resulting in potentially improved gas selectivity and gas adsorption. Molecular simulation results of MOFs having OMSs, particularly Cu‐BTC, resulted in a good agreement with the experimental adsorption data. For instance, Karra and Walton used GCMC simulations to evaluate the adsorption of CO, CH_4_, N_2_, and H_2_ gases in Cu‐BTC,^[^ ^59^ ^]^ and Lamia et al.^[^ ^60^ ^]^ studied the adsorption of C_3_H_8_, C_3_H_6_, and i‐C_4_H_10_ (isobutane) in Cu‐BTC using GCMC simulations, demonstrating good agreement between experimental and simulated adsorption isotherms. Zhao and colleagues used GCMC simulations to study the adsorption of C_6_H_6_ (benzene) in Mg‐MOF‐74 having OMSs and revealed that the simulated isotherms are consistent with experimentally reported results.^[^ ^61^ ^]^ These studies demonstrated the pivotal role of molecular simulations in advancing our understanding of gas adsorption behavior in MOFs, offering valuable insights into the design of better materials.

iii) Revealing the properties of thousands of MOFs through computational screening

In the early molecular simulation studies, MOFs were studied one by one using their crystallographic information files (CIFs) reported in their corresponding synthesis papers. As the number of experimentally synthesized MOFs increases, their CIFs have been deposited into the CSD and researchers aiming to perform molecular simulations downloaded the necessary CIFs from the CSD. The first HTCS study, to the best of our knowledge, in 2010, studied 504 MOFs using the CIFs from the CSD and investigated the membrane‐based H_2_/CH_4_ separation performances of MOFs by combining GCMC and MD simulations.^[^ ^62^ ^]^ Results showed that MOFs can achieve high H_2_ membrane selectivities and permeabilities up to 10^6^ Barrer, outperforming zeolites and polymer membranes for this separation. In 2012, 504 MOFs acquired from the CSD were studied for CO_2_ capture from flue gas, and results showed that MOFs offer great potential by achieving higher CO_2_/N_2_ selectivity (>190) compared to zeolites.^[^ ^63^ ^]^ By screening 1163 MOFs extracted from the CSD, Watanabe and Sholl combined GCMC and MD simulations to calculate CO_2_/N_2_ selectivities of MOF membranes and showed that promising MOF materials could achieve very high membrane selectivities (>800), outperforming traditional polymer membranes.^[^ ^64^ ^]^

The CIFs deposited into the CSD or directly reported in MOF synthesis papers might have some structural problems that pose challenges for performing molecular simulations. These problems can include the existence of solvent molecules in the pores of MOFs, missing hydrogen atoms, or partially occupied or disordered atoms in the framework. Researchers need to perform postmodification on these types of structures and curate each structure manually to make the CIFs accurate and ready for a molecular simulation program, as we will discuss in detail later. This postmodification process was time‐consuming and prone to creating bias due to manual curation. To overcome this bias, save time, and ensure consistency, computation‐ready MOF databases were introduced, offering a set of CIFs for MOFs that have been curated in the same way.

**Figure 2:**
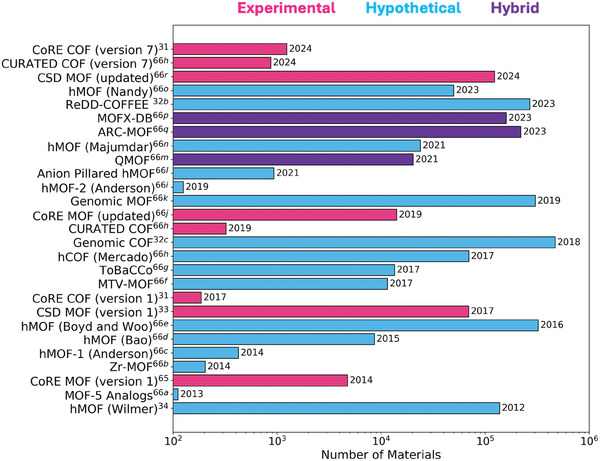
List of publicly available MOF and COF databases that contain >100 structures.^[^ ^31^, ^32^, ^33^, ^34^, ^65^, ^66^ ^]^

In the last decade, there have been massive efforts to generate computation‐ready MOF and COF databases. **Figure** **2** shows the list of publicly released MOF and COF databases containing more than 100 structures from 2012 until today. We categorized them as experimental, hypothetical, and hybrid (including both experimental and hypothetical structures) structure databases. In 2014, Snurr and co‐workers established the first computation‐ready experimental MOF (CoRE MOF) database consisting of over 4700 3D and solvent‐free synthesized MOFs.^[^ ^65^ ^]^ They made the MOFs computation‐ready by removing bound and unbound solvents in the pores, retaining charge‐balancing ions in the cationic/anionic frameworks, and adding missing hydrogen atoms. GCMC simulations of 4764 MOFs were then conducted to compute their deliverable CH_4_ capacities and absolute CH_4_ uptakes. Results showed that the absolute CH_4_ uptake of MIL‐53(Al), 267 cm^3^ STP cm^−3^, exceeded the storage target of the DOE at the time of publication.

In 2017, Jimenez and co‐workers introduced the CSD MOF subset, which is the largest database comprising ≈60 000 synthesized MOFs and encompassing not only 3D structures but also 1D and 2D MOFs.^[^ ^33^ ^]^ The database was supported with a publicly available Python script designed to eliminate bound and unbound solvents, resulting in solvent‐free structures. However, possible issues regarding charge‐balancing ions and missing hydrogen atoms were not addressed, and additional modifications may be required before these structures are implemented in molecular simulations. The CSD MOF subset has been studied for various gas separation applications, including CO_2_/N_2_, CO_2_/CH_4_, CO_2_/H_2_, H_2_/N_2_, and CH_4_/H_2_ separations.^[^ ^67^ ^]^ As shown in Figure 2, the CoRE MOF and CSD MOF databases currently contain 14 142 and 125 383 MOFs, respectively, indicating a rapid growth in the number of synthesized MOFs, more than doubling the number of structures reported in their first versions.

As an alternative to experimentally synthesized MOF databases, Wilmer et al. computationally generated 137 953 MOFs, which is the first publicly available MOF database.^[^ ^34^ ^]^ This hMOF database was screened by GCMC simulations for CH_4_ storage^[^ ^34^ ^]^ and H_2_ storage,^[^ ^68^ ^]^ and results showed several structures can exceed the DOE targets. Bao et al.^[^ ^66d^ ^]^ constructed a database comprising 8629 hMOFs, investigated their CH_4_ storage capabilities, and showed that while high surface area generally correlates with high deliverable capacities, negative correlations can be observed between surface area and deliverable capacities. Snurr and co‐workers generated a database comprising 13 512 hMOFs using the top‐down approach, combining metal nodes and building blocks in certain topological nets, and screened them for CH_4_ and H_2_ storage.^[^ ^66g^ ^]^ Results showed that volumetric and gravimetric CH_4_ and H_2_ uptakes generally depend on the topologies of MOFs because structural properties such as void fraction and surface area are combined differently in different topologies. After 2017, various hMOF databases continued to be released with different purposes. For example, in 2021, Smit and co‐workers^[^ ^66n^ ^]^ aimed to improve the diversity of MOFs and designed an hMOF database (≈20 000 materials) by choosing several metal nodes from the experimentally synthesized MOFs to study them for CO_2_ capture from flue gas and H_2_ storage. Their results showed that using different metal nodes helps in obtaining structures with higher performance compared to the benchmark materials such as zeolite 13X for CO_2_/N_2_ separation and MOF‐5 for H_2_ storage. Perhaps the most challenging issue related to hMOF databases is the unknown structural stabilities of the structures. The majority of hMOF databases do not address the thermal‐ and solvent removal‐stability of the structures. Recently, Kulik and co‐workers constructed an ultrastable hMOF database composed of ≈50 000 structures by using linkers, topologies, and metal nodes of stable, synthesized MOFs and showed that thousands of hMOFs are thermally and mechanically stable, while being suitable for CH_4_ storage.^[^ ^66o^ ^]^

The rapid increase in the number of synthesized and hypothetical MOFs has led to the emergence of hybrid MOF databases. As shown in Figure 2, in 2021, Rosen et al.^[^ ^66m^ ^]^ provided the first hybrid Quantum MOF (QMOF) database, consisting of 20 375 structures with >16 000 experimental MOFs. QMOF is the first publicly available, large database with computed quantum‐chemical properties such as density functional theory (DFT)‐based partial atomic charges and DFT‐based geometry‐optimized structures. Ercakir et al.^[^ ^69^ ^]^ recently screened this database for adsorption‐based n‐butane (C_4_H_10_) capture from air and results showed that over 1000 QMOFs have higher C_4_H_10_ selectivities than a conventional zeolite, MFI. A recent database released in 2023 by Snurr and co‐workers, MOFX‐DB, provided the simulated adsorption isotherms of seven different gas molecules (CO_2_, H_2_, CH_4_, Ar, N_2_, Kr, and Xe) for a total of ≈160 000 hypothetical and synthesized MOFs.^[^ ^66p^ ^]^ To the best of our knowledge, this is the most extensive and easy‐to‐access simulated gas adsorption data reported for MOFs.

Similar to MOFs, COFs have been synthesized and computationally constructed, resulting in various databases. As shown in Figure 2, in 2017, Zhong and co‐workers reported the first computation‐ready and experimental COF database (CoRE COF) comprising 187 structures and focused on their noble gas separation performances.^[^ ^31a^ ^]^ COFs exhibited notable adsorption selectivities for Kr/Ar, Xe/Kr, and Rn/Xe. Smit and co‐workers released the first version of Clean, Uniform, and Refined with Automatic Tracking from the Experimental Database of COFs (CURATED COFs) comprising 324 synthesized structures.^[^ ^31b^ ^]^ CURATED COFs were prepared by performing DFT optimization and assigning DFT‐based partial charges. Today, updated CoRE COF and CURATED COF databases include 1242 and 871 structures, respectively.

Smit and co‐workers released the first hypothetical COF (hCOF) database consisting of 69 840 structures using commercially available linkers and topologies available in the Reticular Chemistry Structure Resource (RCSR).^[^ ^66h^ ^]^ They performed GCMC simulations at a storage pressure of 65 bar, and found that more than 400 hCOFs exceeded the CH_4_ uptake of 280 cm^3^ STP cm^−3^, the DOE target reported for effective CH_4_ storage. Van Speybroeck and co‐workers constructed a new hCOF database consisting of 268 687 materials.^[^ ^32b^ ^]^ They performed GCMC simulations at 5.8 and 65 bar, 298 K to compute the CH_4_ storage capacities of hCOFs and showed that deliverable volumetric capacities are comparable with the best‐reported MOFs. In summary, the establishment of these computation‐ready material databases accelerated the screening efforts for unlocking the full potential of MOFs and COFs for gas storage and separation applications.

iv) Guiding novel material synthesis under the desired conditions

The identification of hMOFs that surpass the performance of existing synthesized ones marks a significant advancement; however, demonstrating their synthesizability is even more crucial for their practical application and relevance as discussed in a recent review.^[^ ^70^ ^]^ For instance, Smit's group screened 325 000 hMOFs to assess their potential for CO_2_/N_2_ separation utilizing GCMC simulations.^[^ ^71^ ^]^ This effort identified 8325 hMOFs with exceptional CO_2_ selectivities (>50) and working capacities (>2 mol kg^−1^). Among these, Al‐PMOF was synthesized and significant CO_2_ uptakes (1.75 mol kg^−1^ (dry) and 1.65 mol kg^−1^ (humid) at 313 K) were experimentally measured, surpassing the traditional adsorbents such as activated carbon and zeolite 13X. To quickly identify an effective MOF for CO_2_ capture from a CO_2_/H_2_ mixture, Wilmer's hMOF subset was filtered by genetic algorithms and GCMC simulations were performed for 730 hMOFs at 20 bar, 313 K.^[^ ^72^ ^]^ CO_2_ working capacities, CO_2_/H_2_ selectivities, and adsorbent performance scores of 730 hMOFs were calculated, and the NOTT‐101/OEt was identified as a promising material with a high CO_2_ working capacity (5.6 mol kg^−1^), outperforming known MOFs. This MOF was then synthesized, and a CO_2_ working capacity of 3.8 mol kg^−1^ was measured, surpassing several well‐studied MOFs. Gómez‐Gualdrón et al.^[^ ^73^ ^]^ constructed 13 512 hMOFs and computed their H_2_ deliverable capacities in between 5 bar, 160 K, and 100 bar, at 77 K by using GCMC simulations. Results revealed that hMOFs can achieve H_2_ deliverable capacities up to 57 g H_2_ L^−1^ and hMOFs with “she” topologies are top‐performing materials. They selected and synthesized four of them (she‐MOF‐x series), and a high H_2_ deliverable capacity (43.4 g H_2_ L^−1^) was measured for she‐MOF‐1, outperforming NU‐1103 (43.2 g H_2_ L^−1^). Ahmed et al.^[^ ^74^ ^]^ utilized GCMC simulations to evaluate 5309 synthesized MOFs from CoRE MOF and Goldsmith's databases for their H_2_ deliverable capacities at a pressure range between 1 and 100 bar, 77 K. They identified four different MOFs to achieve higher usable gravimetric and volumetric H_2_ deliverable capacities than well‐known MOF‐5 (4.5 wt%, and 31.1 g H_2_ L^−1^ in between 5 and 100 bar, 77 K) and synthesized IRMOF‐20 among them to test it experimentally. H_2_ deliverable capacities of IRMOF‐20 were measured as 5.7 wt% and 33.4 g H_2_ L^−1^ in between 5 and 100 bar, 77 K, outperforming MOF‐5. These studies are the pioneer examples of how molecular simulations can guide the synthesis of novel MOFs for given gas adsorption applications, highlighting the importance of enhancing collaborations between experimental and modeling endeavors to design and develop new MOFs for cutting‐edge applications.

### 3.2 Key Inputs for Molecular Simulations

After reviewing the main contributions of molecular simulations to the MOF field, we now visited the key issues that we need to focus on to perform accurate molecular simulations and briefly addressed the critical points in selecting MOF structures, force fields, and gas models.

i) MOF structures: Several software packages, such as Multipurpose Simulation Code (MUSIC),^[^ ^75^ ^]^ Materials Studio,^[^ ^76^ ^]^ and RASPA^[^ ^77^ ^]^ are widely used for molecular simulation of MOFs. To the best of our knowledge, RASPA is the most used molecular simulation software in the field of MOFs. It was developed by Dubbeldam et al. to perform molecular simulations for computing adsorption and diffusion in nanoporous materials.^[^ ^77^ ^]^ The main input of RASPA is the CIFs of MOF structures, which contain important structural details, including atomic positions, bond information, symmetry group, and lattice parameters. The computation‐ready MOF databases that we introduced above report the CIFs of MOFs in a format ready for molecular simulations. There are some important points to consider in using these CIFs in molecular simulations. i) RASPA requires CIFs to be in P1 symmetry but experimentally reported CIFs in the CSD can be reported with symmetries other than P1, so additional steps may be required to convert the structures into P1 symmetry. ii) Some CIFs may not have hydrogen atoms since X‐ray diffraction does not directly reveal their positions, necessitating the addition of these hydrogen atoms into the structure via crystallographic programs. iii) Partial occupancies and disorders can exist within the crystal structures, which require some curation prior to use in molecular simulations. iv) Solvent molecules (bounded and/or unbounded) may be present within the MOF structure. These molecules are typically removed during the activation processes before adsorption experiments, and molecular simulations should ideally replicate this condition to be accurate, implying the necessity to accurately remove the solvent molecules present in the pores. Therefore, computation‐ready databases addressing this issue have been used to study MOFs.

Obtaining the geometric identity of an MOF is essential for the systematic comparison of materials in HTCS studies and to reveal the structure–performance relations. In many HTCS studies focusing on gas adsorption and separation, initial screening steps include the elimination of MOFs based on their pore sizes, such as pore limiting diameter (PLD) or the largest cavity diameter (LCD). The LCD is defined as the largest spherical particle that can be inserted at any point within the material's pores without overlapping with any framework atoms. On the other hand, PLD is defined such that no sphere larger than the structure's limiting diameter can pass through the structure without overlapping with one or more framework atoms. Selecting the MOFs having pore sizes larger than the kinetic diameter of the target guest molecules allows these molecules to be adsorbed into the pores and diffuse through the pores of the framework.

Structural properties of MOFs are generally computed by using CIFs as inputs in software tools like Zeo++^[^ ^78^ ^]^ and Pore Blazer.^[^ ^79^ ^]^ Zeo++ is a C++ package for high‐throughput analysis of porous materials based on the Voronoi tessellation developed by Haranczyk and co‐workers,^[^ ^78^ ^]^ whereas Pore Blazer is a code written in Fortran 90 based on the grid (or lattice) representation of the porous space developed by Fairen‐Jimenez and co‐workers.^[^ ^79^ ^]^ Both tools can calculate the pore volume, accessible gravimetric and volumetric surface areas, pore size such as PLD and LCD, as well as pore size distribution (PSD). Within the RASPA package, there are also options to obtain the pore volume of MOFs and PSD using Monte Carlo simulations. To the best of our knowledge, Zeo++ is the most widely used code to obtain the structural properties of MOFs and one of the most important sources that provides the MOFs’ structural descriptors in machine learning (ML) studies.

ii) Force fields: Force fields are a set of analytical interatomic interaction potentials with an associated set of atomic parameters that describe the interactions between gas–gas and gas–MOFs and have a major influence on the accuracy of molecular simulations. The energy and size parameters of MOF atoms are typically obtained using generic force fields, mostly the universal force field (UFF)^[^ ^80^ ^]^ and the DREIDING^[^ ^81^ ^]^ force field, which were developed for systems including organic compounds and the optimized potential for the liquid simulations of all‐atom (OPLS‐AA)^[^ ^82^ ^]^ force field which was developed for liquid simulations. Parameters of octahedral copper and zinc atoms, which are common building blocks of MOFs, are not present in UFF. Therefore, 18 different metal atom types were added to construct^[^ ^83^ ^]^ UFF specific for MOFs (UFF4MOF),^[^ ^84^ ^]^ which includes the parameters that are missing in UFF.

Highly precise force fields known as ab initio force fields were developed for studying the gas adsorption properties of a group of MOFs. For instance, Schmid and colleagues developed an MOF‐based force field (MOF‐FF) by integrating genetic algorithms and ab initio methods.^[^ ^85^ ^]^ This approach aimed to define more precise force field parameters for well‐known MOFs, such as IRMOF‐1 and Cu‐BTC, compared to the generic force fields. Evans et al.^[^ ^86^ ^]^ used this force field in GCMC simulations to compute CH_4_ adsorption in DUT‐49. Results showed that the usage of this force field validates the phenomena of negative gas adsorption in this MOF. Vanduyfhuys et al.^[^ ^87^ ^]^ developed an ab initio force field, QuickFF, which derives system‐specific force fields from the ab initio computed Hessian matrix, which was used to describe the negative gas adsorption phase transition in DUT‐49, including the flexibility of the framework during molecular simulations.^[^ ^88^ ^]^ Deng and co‐workers derived an accurate van der Waals force field (VDW FF) from quantum mechanical calculations to accurately compute H_2_, CO_2_, and CH_4_ adsorption in MOFs and COFs.^[^ ^89^ ^]^ Their findings demonstrated that the results obtained using GCMC simulations with VDW FF were closer to the experimentally measured uptake values compared to those obtained with general force fields, UFF and DREIDING. Prakash et al.^[^ ^90^ ^]^ developed an ab initio‐based force field and computed H_2_, N_2_, and CH_4_ adsorptions in ZIF‐95 and ZIF‐100 through GCMC simulations. Results unlocked several fundamentals related to gas adsorption mechanisms for these materials.

Accurate definition of intermolecular interactions between gas molecules and MOFs with unique features such as OMSs or flexibility poses significant challenges. To address these issues, new force fields were developed. For example, Sholl and co‐workers developed an ab initio force field to address the chemical interactions between water molecules and open Cu sites in Cu‐BTC.^[^ ^91^ ^]^ Results showed a good agreement between experimental and simulated isotherms not only for Cu‐BTC but also for methyl‐ and ethyl‐functionalized (Cu‐MBTC and Cu‐EBTC) versions, where they transferred the force field. Haldoupis et al.^[^ ^92^ ^]^ developed an ab initio force field for the M‐MOF‐74 (M = Mn, Co, Ni, and Cu) series and demonstrated the good agreement between experimental and simulated CO_2_ adsorption isotherms. Gagliardi and co‐workers developed an ab initio force field to study CO_2_ adsorption in Mg‐MOF‐74.^[^ ^93^ ^]^ Results showed that simulations employing the newly developed force field yielded more consistent results with the experimental findings compared to those obtained from the UFF. A DFT‐based force field was developed to examine CO_2_ adsorption in flexible ZIF‐8 and ZIF‐71.^[^ ^94^ ^]^ GCMC simulations were conducted by employing both this force field and UFF to calculate the CO_2_ uptakes of the MOFs. Results demonstrated that the simulation results utilizing the DFT‐based force field are significantly closer to the experimental values compared to those derived from UFF. Smit and co‐workers developed accurate force fields from DFT calculations to predict single‐component CO_2_ and H_2_O adsorption and CO_2_/H_2_O mixture adsorption data in MOFs with OMSs, Mg‐MOF‐74, and Zn‐MOF‐74.^[^ ^95^ ^]^ They showed that GCMC calculations with this force field provide closer gas uptakes to the experimental results than those using the UFF. Chen et al.^[^ ^96^ ^]^ presented an approach for predicting CH_4_ adsorption in MOFs with OMSs by directly implementing ab initio derived potential energy surface (PES) in GCMC simulations. This method addresses the limitations of generic force fields, which often fail to accurately describe interactions at OMSs, by using hybrid DFT/ab initio calculations to create a more precise PES. The study demonstrated that the proposed approach using Cu‐BTC not only quantitatively predicted adsorption isotherms over a range of temperatures and pressures but also accurately captured the adsorption mechanism. A hybrid computational approach for modeling interactions between OMSs in MOFs and adsorbate molecules has been used to integrate DFT calculations to accurately describe the specific interactions at the OMS while performing GCMC simulations for computing gas adsorption to overcome the limitations of generic force fields.^[^ ^97^ ^]^ Fischer et al.^[^ ^98^ ^]^ used this approach for accurately predicting H_2_ adsorption in MOFs with OMSs. The authors derived new potential parameters for the H_2_–metal interaction from ab initio calculations and integrated these into GCMC simulations. Results showed that the used approach significantly improved the prediction of low‐pressure H_2_ adsorption isotherms in MOFs like Cu‐BTC and PCN‐12, while the generic force‐field parameters perform better at higher pressures. All these studies show that the usage of the most appropriate force field is a key factor for accurate molecular simulations, as it defines the interactions between the host and adsorbate. Generic force fields such as UFF and DREIDING can work very well for simple systems where the MOFs are rigid and they do not have OMSs, however for more complex systems where MOFs are flexible and having OMSs, using tailored DFT‐based force fields is a better choice.

Another point related to MOF structures together with the force fields is to assign partial atomic charges to framework atoms. Partial atomic charges are not directly reported in CIFs deposited into the CSD but are needed to perform simulations for guest molecules having dipole or quadrupole moments, such as CO_2_ and H_2_O, to compute their electrostatic interactions with the MOFs. Various methods differing in complexity and time‐efficiency exist to compute atomic charges as discussed by Smit and co‐workers.^[^ ^99^ ^]^ One type of DFT‐based charge methods is based on fitting the electrostatic potential around the molecular structure. The Charges from Electrostatic Potentials using a Grid (CHELPG) method^[^ ^100^ ^]^ is used to determine partial atomic charges by fitting them to reproduce the molecular electrostatic potential. This method involves calculating the electrostatic potential at a series of points around the molecule, typically arranged on a 3D grid, and then adjusting the atomic charges to best match the calculated potential at these grid points. The Repeating Electrostatic Potential Extracted Atomic Charges (REPEAT) method^[^ ^101^ ^]^ is designed to generate atomic charges that reproduce the electrostatic potential derived from DFT calculations. This method involves performing periodic DFT calculations to obtain the electrostatic potential of the entire system and then fitting atomic charges to match this potential. Another type of DFT‐based charge assignment is orbital‐based methods. Mulliken method^[^ ^102^ ^]^ assigns atomic charges based on the overlap of atomic basis functions in the molecular orbitals, but it is highly dependent on the choice of basis set. In contrast, Natural Population Analysis (NPA) uses Natural Bond Orbitals (NBO) to partition electron density into natural atomic orbitals, yielding more stable and chemically meaningful charges that are less sensitive to the basis set.^[^ ^103^ ^]^ The third type of DFT‐based charge assignment methods is based on partitioning the electron density. Partitioning electron density methods, such as those in the Hirshfeld family,^[^ ^104^ ^]^ distribute a molecule's electron density among its atoms to assign atomic charges accurately. The basic Hirshfeld method uses reference densities from isolated atoms to proportionally assign electron density. This generally results in relatively low charges that often underestimate the electrostatic potential around each atom, prompting the development of various improved methods over the years. The Hirshfeld method may not always accurately reflect the electronic environment in highly polar or charged systems. Therefore, new methods have emerged to overcome these difficulties. Van Speybroeck and colleagues developed the Minimal Basis Iterative Stockholder (MBIS) method to generate accurate atomic charges by iteratively fitting to the electrostatic potential derived from ab initio calculations.^[^ ^105^ ^]^ The iterative nature of the MBIS method ensures that the resulting atomic charges are consistent with the overall molecular electrostatic potential, providing a robust and accurate description of the electrostatic interactions within MOFs. Density‐derived electrostatic and chemical (DDEC) charges^[^ ^106^ ^]^ combines aspects of Hirshfeld and electrostatic potential‐based methods to provide charges that reproduce the electrostatic potential around the structure more accurately. Until 2021, there was only one publicly available MOF subset^[^ ^107^ ^]^ (≈2900 structures from the CoRE MOF 2014 database) with DDEC charges. In 2021, the QMOF database^[^ ^66m^ ^]^ provided the CIFs with DDEC charges and in 2023, ab initio REPEAT charge MOF (ARC‐MOF) database, was released with ≈221 000 structures with their DFT‐based partial atomic charges.^[^ ^66q^ ^]^

Determining point charges through DFT‐based approaches can require significant computational time and resources. With the advent of HTCS of MOFs, there is a pressing need for methods that can produce chemically reasonable charges with lower computational demands. As a result, various versions of approximate charge equilibration (Qeq) methods with different versions have been widely used in the HTCS of MOFs. Qeq method^[^ ^108^ ^]^ predicts charge distributions by using measurable properties, including atomic ionization potential energies, electron affinities, and atomic radii. This method constructs an atomic chemical potential that, when equalized across all atoms, leads to equilibrium charges dependent on molecular geometry. The periodic charge equilibration (PQEq) method^[^ ^109^ ^]^ is an extension of the Qeq method designed to account for the polarizability of atoms. PQEq introduces a dynamic aspect by allowing charges to polarize in response to the electrostatic environment. The extended charge equilibration (EQeq)^[^ ^110^ ^]^ method is an enhancement of the Qeq method, aiming to improve the accuracy of charge distributions in more complex molecular systems by integrating additional factors, such as higher ionization energies, to better account for the dynamic nature of charge distribution. The MOF electrostatic potential optimized charge equilibration (MEPO‐Qeq) method^[^ ^111^ ^]^ is a parametrization of the Qeq method specifically tailored for MOFs. This method optimizes the Qeq parameters to reproduce the electrostatic potential derived from DFT calculations for a diverse set of MOFs. The MEPO‐Qeq method ensures that the generated partial atomic charges closely mimic the ab initio electrostatic potential, enabling accurate predictions of nonbonded electrostatic interactions. To the best of our knowledge, there is no publicly available database that directly provides the approximate partial charges. Researchers can use the Qeq method as implemented in the RASPA if their structures are not reported with the DFT‐based charges. Recently, ML algorithms, trained on a very large collection of DDEC charges, were shown to accurately and quickly assign such charges for MOF atoms and published as a Python library, PACMOF.^[^ ^112^ ^]^ It has been shown that the choice of charge assignment method can result in significant variations in the charges obtained and, more critically, in the resulting isotherms.^[^ ^113^ ^]^ Therefore, these developments have been very useful in achieving fast, accurate, and consistent charge calculations, one of the critical steps in molecular simulations of MOFs, because of their impact on the final performance assessment.^[^ ^114^ ^]^

iii) Guest molecules: As our review focuses on the adsorption of four guest molecules (CO_2_, CH_4_, H_2_, and H_2_O) in MOFs, we specifically addressed their models utilized in molecular simulations. TraPPE^[^ ^115^ ^]^ and EPM2^[^ ^116^ ^]^ models were developed to mimic the vapor–liquid phase behavior of CO_2_ molecules and are widely used in molecular simulations. Both models represent CO_2_ as a linear, three‐site rigid molecule with point charges located at the center of each site. In many simulation studies,^[^ ^66^, ^117^ ^]^ CH_4_ has been modeled as a nonpolar, single, and spherical atom.^[^ ^118^ ^]^ However, an alternative representation includes a five‐site model where all atoms are explicitly considered.^[^ ^119^ ^]^ H_2_ is commonly represented as a nonpolar, single, and spherical atom using the Buch model,^[^ ^67^, ^120^ ^]^ a two‐site model,^[^ ^121^ ^]^ and a three‐site model^[^ ^122^ ^]^ in many HTCS studies. For H_2_O, there are several HTCS studies focusing on the effect of H_2_O adsorption on the separation performance of MOFs and understanding its adsorption mechanism.^[^ ^67^, ^123^ ^]^ In these studies, the TIP4P model,^[^ ^124^ ^]^ which represents H_2_O with a four‐site model and nonpolarizable atoms, was widely employed. Another alternative is TIP4P/2005, which was also compared with the TIP4P model for hydrophobic ZIF‐8 and Zn‐MOFs.^[^ ^125^ ^]^ Results showed the good agreement between simulated and experimental isotherms when the TIP4P model was employed. Overall, the selection of the gas model holds significance as it influences the results of molecular simulations.

## 4 Applications of MOFs

**Figure 3:**
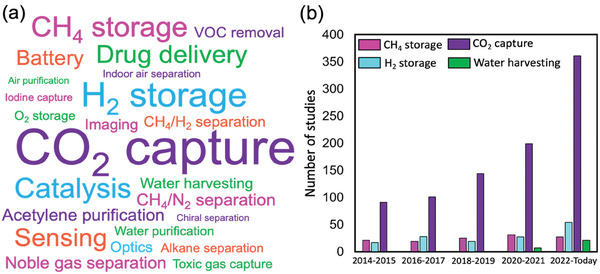
a) Word cloud illustration for MOF applications studied through molecular simulations. The sizes of the words indicate the frequency of their appearance in publications. Scopus‐based literature survey was conducted on April 5, 2024, using the keywords “metal–organic framework” or “MOF” and “simulation.” This research has yielded a total of 3782 simulation studies that have been published since 2000. b) The number of computational studies focusing on CO_2_ capture, CH_4_ storage, H_2_ storage, and water harvesting since 2014.

In this section, we focused on computational studies for a variety of gas adsorption applications of MOFs. To represent the most widely studied applications of MOFs, we created a word cloud graph, **Figure** **3**a, by conducting a search on Scopus using “metal–organic framework” or “MOF” and “simulation” keywords. Between 2000 and 2024, a total of 3782 articles used molecular simulations to study MOFs. Among these, 1084 articles focus on CO_2_ capture, 225 on H_2_ storage, 150 on CH_4_ storage, and 96 on water harvesting. We observed a growing trend in the computational studies for these four applications in recent years, as presented in Figure 3b since the design and discovery of novel materials for clean air, clean energy, and clean water applications are getting more and more crucial. For instance, the number of computational studies focusing on CO_2_ capture increased from 199 in 2020–2021 to 361 in 2022–today. There are several studies that screened hundreds of MOFs for these applications, and we discuss them in detail below.

### 4.1 CO_2_ Capture

CO_2_ adsorption is critical to combat climate change by reducing greenhouse gas emissions.^[^ ^126^ ^]^ Capturing CO_2_ from various sources, such as industrial processes and power plants helps mitigate global warming and its detrimental effects on the environment, ecosystems, and human health by preventing CO_2_ from entering the atmosphere.^[^ ^127^ ^]^ Molecular simulation studies of MOFs for CO_2_ capture primarily focused on capturing CO_2_ mainly from N_2_ and CH_4_, whereas only a few studies addressed the storage of pure CO_2_.^[^ ^128^ ^]^

#### 4.1.1 Flue Gas Separation

Flue gas, predominantly composed of CO_2_ and N_2_, is generated from combustion processes in power plants and industrial facilities. Carbon capture and storage (CCS) technologies heavily rely on efficient flue gas separation methods to sequester CO_2_. MOFs have been widely studied for CO_2_/N_2_ adsorption and separation thanks to their high CO_2_ affinities. Demir and Keskin used GCMC simulations to calculate CO_2_/N_2_ mixture adsorption data of 936 anion‐pillared MOFs to evaluate their separation potentials.^[^ ^129^ ^]^ Results showed that most CO_2_ selective MOFs achieve CO_2_ selectivities between 84.3 and 708.4 at 1 bar, 298 K, and have narrow pores (<5 Å) and small surface areas (<750 m^2^ g^−1^). Qiao et al.^[^ ^130^ ^]^ screened 4764 CoRE MOFs by using GCMC simulations for the CO_2_/N_2_ mixture. Selectivity, working capacity, and regenerability were used to assess the MOFs and materials containing alkali and alkaline metals were shown to have high selectivity and working capacity but low regenerability. Lanthanide‐based MOFs exhibited the highest CO_2_/N_2_ selectivities (1.1 × 10^3^–3.2 × 10^4^) at 0.1, 1, and 10 bar, 298 K despite their weaker representation compared to alkali and alkaline‐based MOFs in the CoRE MOF database.

**Figure 4:**
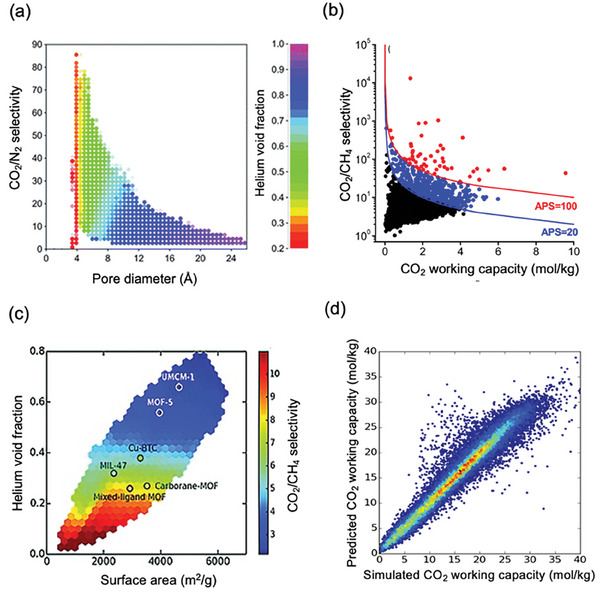
a) Relationship between CO_2_/N_2_ selectivity and pore diameter over 130 000 hMOFs at 298 K, 1 bar. Adapted with permission.^[^ ^132^ ^]^ Copyright 2012, Royal Society of Chemistry. b) CO_2_/CH_4_ selectivity, CO_2_ working capacity, and adsorbent performance score (APS) of 3816 CSD MOFs computed at 298 K and an adsorption (desorption) pressure of 1 (0.1) bar for an equimolar CO_2_/CH_4_ mixture. Reproduced under the terms of the Creative Commons Attribution (CC‐BY) license.^[^ ^67a^ ^]^ Copyright 2018, American Chemical Society. c) Scatter plots of the helium void fraction and surface area of 324 500 MOFs. Color bar represents the CO_2_/CH_4_ selectivity of structures. Reproduced with permission.^[^ ^134^ ^]^ Copyright 2016, Wiley‐VCH. d) Heatmaps of simulated CO_2_ working capacity against ML‐predicted CO_2_ working capacity for 35 840 hMOFs at adsorption and desorption pressures of 40 and 1 bar, respectively, at 313 K. The colors of the heatmaps correspond to the number of MOFs, where red is high, and blue is low. Reproduced with permission.^[^ ^135^ ^]^ Copyright 2019, American Chemical Society.

Leperi et al.^[^ ^131^ ^]^ screened 5109 CoRE MOFs through the utilization of GCMC simulations in conjunction with a fractionated vacuum pressure swing adsorption model. Their analysis identified 190 MOFs that achieved the desired criteria for effective CO_2_/N_2_ separation, specifically exceeding 90% in both purity and recovery targets. Snurr and co‐workers constructed 10 995 multivariate MOFs (MTV‐MOFs) and utilized GCMC simulations for computing their CO_2_/N_2_ mixture adsorption data at 1 bar, 298 K.^[^ ^66f^ ^]^ Results revealed that functionalized and narrow‐pored (<7 Å) MOFs achieve higher CO_2_ capacities up to 6.5 mol kg^−1^, and CO_2_/N_2_ selectivities up to 35, outperforming nonfunctionalized ones. Mohamed et al.^[^ ^66q^ ^]^ used 15 412 hMOFs from the ARC‐MOF database for which the GCMC simulation data was available for CO_2_/N_2_ separation at 0.9 bar, 298 K, and combined them with several stability metrics to identify the top‐performing stable hMOFs. Results revealed that 51 hMOFs achieve CO_2_ uptakes with >4 mol kg^−1^ and CO_2_/N_2_ selectivities >200 and these materials are thermally, mechanically, and chemically stable. Snurr and co‐workers also examined over 130 000 h MOFs by employing molecular simulations to assess their CO_2_/N_2_ separation performances.^[^ ^132^ ^]^ Results showed that materials with high CO_2_/N_2_ selectivities have narrow pore diameters (<8 Å) and low void fractions (<0.6), as shown in **Figure** **4**a. They also discovered that specific functional groups, particularly those containing fluorine and chlorine atoms, are commonly associated with superior CO_2_/N_2_ separation performance.

While most research have concentrated on separating CO_2_ from N_2_, flue gas contains additional impurities such as SO*_x_*, NO*_x_*, and H_2_O. The presence of water vapor can significantly impact the separation performance of MOFs as water molecules can compete with CO_2_ for the available adsorption sites. To assess the effect of humidity on the CO_2_ capture performance of MOFs, Li et al.^[^ ^123b^ ^]^ screened the CoRE MOF database, eliminating MOFs with lower H_2_O adsorption energies than the heat of vaporization of water. They calculated CO_2_/H_2_O selectivities based on the ratio of Henry's constants to identify the most CO_2_‐selective MOFs. The top MOFs were then evaluated for CO_2_/H_2_O/N_2_ separation, which demonstrated high selectivity toward CO_2_ despite the presence of H_2_O and N_2_. Kancharlapalli and Snurr utilized a multiscale screening approach to identify the adsorbents with high CO_2_/N_2_ selectivities in the CoRE MOF database.^[^ ^133^ ^]^ They integrated GCMC simulations with DFT calculations and identified 20 promising MOFs with strong CO_2_ binding energy compared to H_2_O and performed simulations at mixture conditions.

In recent years, HTCS and ML have been integrated to rapidly investigate the very large MOF materials space and to identify the hidden structural and chemical patterns. Gómez‐Gualdrón and co‐workers^[^ ^66c^ ^]^ investigated the impact of pore chemistry and topology on the CO_2_/N_2_ separation performances of 400 hMOFs by integrating molecular simulations with ML algorithms. Results highlighted that functionalizing MOFs with thiol, cyano, amino, and nitro groups often improved their CO_2_ capture performance, demonstrating the critical role of pore chemistry. Woo and co‐workers integrated molecular simulations, process simulations, and ML algorithms to screen 1584 CoRE MOFs together with 32 well‐known MOFs, 8 zeolites, and 8 porous polymer networks (PPNs) for the separation of CO_2_/N_2_ mixture at vacuum swing adsorption (VSA) condition.^[^ ^136^ ^]^ They utilized GCMC simulations to compute mixture gas adsorption isotherms of CO_2_ and N_2_ at 298 K and between 0.01 and 1.2 bar for all materials and used this data to develop ML algorithms identifying 482 MOFs to meet the 95% CO_2_ purity and 90% CO_2_ recovery targets for a VSA process. Deng et al.^[^ ^137^ ^]^ integrated molecular simulations and ML algorithms to evaluate CO_2_/N_2_ separation performances of 6013 CoRE MOFs with the presence of impurity, O_2_. They identified that top‐performing MOFs achieve CO_2_/(N_2_ + O_2_) selectivities between 6.3 and 4712, as they tend to have narrow pores (<6 Å) and high heat of adsorption for CO_2_ (26–52 kJ mol^−1^). Woo and co‐workers used GCMC simulations to compute CO_2_/N_2_ mixture adsorption data of 342 489 hMOFs at 0.03 and 1 bar, 298 K, and constructed ML algorithms utilizing this data.^[^ ^138^ ^]^ They discovered that hMOFs can achieve CO_2_ working capacities up to 4.5 mol kg^−1^ and CO_2_/N_2_ selectivities up to 500.^[^ ^138^ ^]^ Lee and co‐workers integrated GCMC simulations and ML algorithms to discover hydrophobic and highly CO_2_‐selective MOFs among 405 hMOFs for CO_2_/N_2_ separation at 1 and 10 bar, 298 K.^[^ ^139^ ^]^ They identified the top‐performing and hydrophobic hMOFs achieving CO_2_/N_2_ selectivities of 22–37, and CO_2_ working capacities changing in between 3.9 and 5.6 mol kg^−1^.

#### 4.1.2 Natural Gas Purification

Separation of CO_2_ from CH_4_ is vital for optimizing the quality and value of natural gas, essential for industrial processes. Several HTCS studies have been conducted to examine the CO_2_/CH_4_ separation capabilities of MOFs. Wilmer et al.^[^ ^132^ ^]^ conducted the first HTCS study by evaluating over 130 000 hMOFs for the separation of an equimolar CO_2_/CH_4_ mixture. They used GCMC simulations to calculate CO_2_ and CH_4_ uptakes at 0.1, 1, and 5 bar, 298 K and mimic VSA and pressure swing adsorption (PSA) processes. They revealed that hMOFs exhibit the heat of adsorption values for CO_2_ around 20 kJ mol^−1^, gravimetric surface areas in between 1000–2000 m^2^ g^−1^ and fluorine functional groups tend to become the top‐performing adsorbents. Altintas and Keskin screened 1500 CoRE MOFs to evaluate their CO_2_/CH_4_ separation potentials at 0.1, 1, and 5 bar, 298 K to examine how different charge assignment methods affect the results of GCMC simulations.^[^ ^114^ ^]^ Results revealed that top‐performing MOFs achieve selectivities between 2.5 and 318 (2.6 and 363) when the DDEC (Qeq) method was used. Rogacka et al.^[^ ^123a^ ^]^ examined the CO_2_/CH_4_ separation performances of 2932 CoRE MOFs at 1 bar, 298 K and identified top‐performing MOFs with CO_2_/CH_4_ selectivities up to 230, featuring zinc ions, nitrogen atoms, and narrow channels (<8 Å). Altintas et al.^[^ ^67a^ ^]^ performed GCMC simulations to investigate the CO_2_/CH_4_ separation performances of a subset of 3816 MOFs acquired from the CSD MOF database at 0.1, 1, and 10 bar, 298 K. Their findings highlighted that MOFs display high selectivities (up to 1000), working capacities (up to 9.6 mol kg^−1^), and adsorbent performance scores (up to 4000 mol kg^−1^) for CO_2_/CH_4_ separation, as illustrated in Figure 4b. Several MOFs were reported to have significantly higher CO_2_/CH_4_ selectivities and CO_2_ working capacities (43–56 and >5 mol kg^−1^) than those of traditional zeolites, MFI (2.8 and 0.7 mol kg^−1^) and 13X (8.3 and 2.7 mol kg^−1^).

Besides CO_2_, natural gas also contains other impurities, such as H_2_S and H_2_O. Siepmann and co‐workers conducted GCMC simulations for 5109 CoRE MOFs for the separation of biogas with small amounts of impurities (50% CH_4_, 45% CO_2_, 3% N_2_, 1% H_2_S, and 1% NH_3_).^[^ ^140^ ^]^ The top MOFs demonstrated high CO_2_/CH_4_ selectivities (up to 330) with a large range of regenerability (15–88%) and working capacity (0.03–2.9 mol kg^−1^). Qiao et al.^[^ ^141^ ^]^ screened 6103 CoRE MOFs for the separation of natural gas composed of six different components, including C_2_H_6_, C_3_H_8_, H_2_S, and H_2_O. Focusing on 606 hydrophobic MOFs, they identified 45 top‐performing MOFs based on selectivity and working capacity, with 39 featuring N‐containing organic linkers, 23 of them containing pyridines, and 12 of them containing azoles.

ML and molecular simulations have been recently integrated to accelerate the discovery of promising MOFs for natural gas purification. Zhao and co‐workers computed CO_2_/H_2_S/CH_4_ mixture adsorption data of 2932 CoRE MOFs by integrating GCMC simulations and ML algorithms at 0.1 and 7 bar, 298 K.^[^ ^142^ ^]^ They computed (CO_2_ + H_2_S)/CH_4_ selectivities of the top‐performing MOFs between 33.5 and 608 and CO_2_ + H_2_S regenerabilities between 70.1% and 98.6%. Their findings revealed that the best materials have narrow pores (5–7.5 Å) and mediocre porosities (0.31–0.77). Aspuru‐Guzik and co‐workers constructed 45 000 hMOFs to discover highly CO_2_‐selective ones for CO_2_/CH_4_ separation.^[^ ^143^ ^]^ They utilized GCMC simulations to compute their CO_2_/CH_4_ mixture adsorption data at 5 bar, 300 K, and revealed that top‐performing hMOFs can achieve high CO_2_ uptakes (up to 7.5 mol kg^−1^) and CO_2_/CH_4_ selectivities (16.0), outperforming well‐known MOFs such as IRMOF‐1, ZIF‐8, and Cu‐BTC. Woo's group utilized GCMC simulations to compute CO_2_/CH_4_ mixture adsorption data of more than 320 000 hMOFs and calculated their CO_2_/CH_4_ selectivities and CO_2_ working capacities.^[^ ^134^ ^]^ Figure 4c shows that CO_2_/CH_4_ selectivities tend to decrease when the surface area, pore diameter, and void fraction exceed 4000 m^2 g−1^, 10 Å, and 0.4, respectively. Conversely, MOFs with lower surface area, pore diameter, and void fraction displayed selectivity higher than 5. Results also indicated that several hMOFs outperform well‐known MOFs such as CuBTC, MOF‐5, UMCM‐1, and MIL‐47, as presented in Figure 4c. They also trained quantitative structure–property relationship (QSPR) models by using the simulation data of 32 500 MOF structures and demonstrated that the models could accurately identify up to 90% of top‐performing hMOFs determined by the results of molecular simulations. All these studies showed that by integrating molecular simulations with ML, researchers can efficiently screen very large libraries of MOFs, identify promising candidates that outperform well‐known adsorbents, and extract the most important chemical and structural properties of MOFs that lead to high CO_2_ selectivity.

#### 4.1.3 Syngas Separation

The primary constituents of the syngas, produced after steam reforming of methane, are CO_2_ and H_2_, representing the most economically viable method for hydrogen generation globally.^[^ ^144^ ^]^ MOFs have emerged as promising materials that can accomplish CO_2_/H_2_ separation with high performance, and several HTCS studies focused on pinpointing the most optimal MOF adsorbents for this separation. Avci et al.^[^ ^67c^ ^]^ conducted the first HTCS study for CO_2_/H_2_ separation by utilizing GCMC simulations to compute mixture adsorption data of 3857 MOFs collected from the CSD. Top‐performing MOFs achieved CO_2_/H_2_ selectivities and APS in between 154.7–484.5 (557.3–2402) and 1289–2365 (1442–6339) mol kg^−1^, respectively, at PSA (VSA) condition, outperforming well‐known zeolites, NaX, and NaY. The same research group collected and screened 10221 MOFs from the updated CSD for CO_2_/H_2_ separation by using GCMC simulations at PSA, VSA, and temperature swing adsorption (TSA) conditions.^[^ ^145^ ^]^ They showed that newly added MOFs to the CSD MOF subset have 15 times higher CO_2_/H_2_ selectivities and 1.3 times higher CO_2_ working capacities compared to those acquired for the MOF subset in their previous study. Wu and co‐workers adopted HTCS approach to discover new MOF adsorbents among 13 512 hMOFs acquired from the Topologically Based Crystal Constructor (ToBaCCo) database for efficient CO_2_/H_2_ separation at 10 bar, 298 K.^[^ ^121b^ ^]^ Top‐performing MOFs were computed to have CO_2_/H_2_ selectivities up to ≈600, and CO_2_ capacities up to ≈25 mol kg^−1^. Dureckova et al.^[^ ^135^ ^]^ integrated GCMC simulations with QSPR models to screen ≈358 400 hMOFs to identify the best MOF adsorbents for CO_2_/H_2_ separation at 1 and 40 bar, 313 K. They showed the good agreement between simulated and predicted CO_2_ working capacities for the 35 840 hMOFs in the test set, as shown in Figure 4d. The majority of hMOFs exhibited CO_2_ working capacities between 10 and 20 mol kg^−1^, and no MOFs possessed both low CO_2_ working capacity and low selectivity. Most MOFs with low working capacity (<5 mol kg^−1^) exhibited CO_2_/H_2_ selectivities greater than 40. Results also demonstrated that QSPR models could identify the top 1000 high‐performing MOFs among the top 5000 MOFs determined by GCMC simulations.

#### 4.1.4 CO_2_ Storage

Simulation studies in the literature concerning CO_2_ storage have addressed only a small number of MOFs. In one of the earliest simulation works, Chen and co‐workers studied the CO_2_ storage capabilities of nine MOFs (IRMOFs ‐1, ‐8, ‐10, ‐11, ‐14, and ‐16, Mn‐MOF, MOF‐177, and Cu‐BTC) up to 60 bar, 298 K.^[^ ^146^ ^]^ Results showed that MOFs possessing a pore size ranging from 10 to 20 Å exhibit high CO_2_ adsorption capacities. Karra and Walton examined CO_2_ adsorption in four MOFs (IRMOF‐1, IRMOF‐3, Cu‐BTC, Zn_2_[bdc]_2_[dabco]) up to 45 bar, 298 K and showed that MOFs with smaller pores can have similar impacts on the CO_2_ adsorption to those with larger pores and OMSs.^[^ ^52^ ^]^ The higher adsorption of CO_2_ in IRMOF‐3 compared to IRMOF‐1 at lower pressures were attributed to amine‐functionalized groups and the slightly smaller pore size of IRMOF‐3. Babarao and Jiang employed GCMC simulations to determine the CO_2_ storage capacities of nine MOFs and one COF up to 50 bar and 298 K.^[^ ^147^ ^]^ Their study highlighted the vital role of the organic linker in achieving high CO_2_ adsorption. The organic linkers of IRMOF‐10 and IRMOF‐14 exhibit greater length and size compared to those present in IRMOF‐1, resulting in increased free volume and accessible surface area, thereby enhancing CO_2_ adsorption as pressure increases.

Identifying the most effective functional groups to enhance the CO_2_ adsorption capacity of MOFs is challenging due to the existence of several functional groups that can be used. An ML algorithm was developed to identify 1035 functionalized MOFs with high CO_2_ uptakes (>3 mmol g^−1^) derived from 141 MOFs and 26 functional groups using the results of GCMC simulations.^[^ ^148^ ^]^ Optimizing functional groups led to an average 3.7‐fold increase in CO_2_ capacities across 141 MOFs, with functionalized versions of extensively studied MOFs like MIL‐47, HKUST‐1, and UiO‐67 exhibiting significantly high CO_2_ uptakes (up to 4.6 mol kg^−1^). Fernandez et al.^[^ ^149^ ^]^ developed a comprehensive QSPR model using atomic property weighted radial distribution function (AP‐RDF) features to predict the CO_2_ storage capabilities of 324 500 hMOFs based on simulated CO_2_ uptakes. The QSPR model distinguished between high‐performing MOFs, which exhibited CO_2_ uptakes exceeding 1 mol kg^−1^ at 0.15 bar and 4 mol kg^−1^ at 1 bar, and low‐performing ones. The model successfully identified 945 high‐performing MOFs (with CO_2_ uptake capacity >1 mol kg^−1^) out of the top 1000 MOFs determined from the results of GCMC simulations.

Moosavi et al.^[^ ^150^ ^]^ utilized GCMC simulations to compute CO_2_ capacities of over 20 000 synthesized MOFs and hMOFs from CoRE MOF and Boyd‐Woo databases at 0.15 and 16 bar, 298 K and used the simulation data to construct ML algorithms with revised autocorrelation functions (RACs), which are products or differences of properties such as electronegativity, nuclear charge, atom identity, connectivity, covalent radii, and polarizability to define the chemistry of MOFs.^[^ ^83^ ^]^ Results demonstrated that pore geometry has emerged as the most influential property in the Boyd‐Woo database, whereas metal chemistry was highlighted in the CoRE MOF database. Overall, ML methods and new descriptors such as AP‐RDF and RACs have significantly improved the ability to predict the gas adsorption properties of MOFs and identify high‐performing candidates, offering valuable insights for their potential applications in CO_2_ storage and separation processes.

### 4.2 CH_4_ Storage

Methane is a major component of natural gas and is a clean alternative to oil. Since it has a very low energy density at room temperature and pressure, it needs to be compressed or liquefied, which makes it difficult to integrate into vehicles. The Advanced Research Projects Agency‐Energy (ARPA‐E) set a target that an adsorbent material should deliver 315 cm^3^ STP cm^−3^ of CH_4_ to the engine using adsorbed natural gas (ANG) conditions a storage pressure of 65 bar and a delivery pressure of 5.8 bar at 298 K.

**Figure 5:**
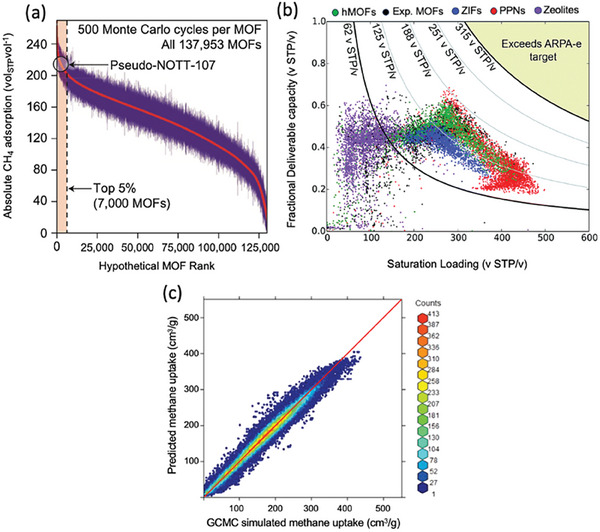
a) Screening of 137 953 MOFs for CH_4_ storage at 35 bar through molecular simulations and selecting one of the promising MOF (NOTT‐107) to synthesize. Reproduced with permission.^[^ ^34^ ^]^ Copyright 2012, Springer Nature. b) Fractional deliverable capacity plotted against the saturation loading for each porous material. Hyperbolas indicate lines of constant deliverable capacity. Reproduced with permission.^[^ ^154^ ^]^ Copyright 2015, Royal Society of Chemistry. c) Parity plots for ML‐predicted versus GCMC simulated CH_4_ uptake using structural and chemical variables. The color scale indicates the number of hMOFs that had the corresponding GCMC and ML results. Reproduced with permission.^[^ ^155^ ^]^ Copyright 2017, American Chemical Society.

The first large‐scale computational screening study of MOFs for CH_4_ storage was performed by Wilmer et al. in 2012.^[^ ^34^ ^]^ GCMC simulations were performed for 137 953 hMOFs for calculating CH_4_ uptakes at 35 bar, 298 K, and more than 300 hMOFs achieved high CH_4_ uptake capacities (>230 cm^3^ STP cm^−3^). Based on the GCMC results, they selected and synthesized one of the promising MOFs, named as NOTT‐107, and measured its CH_4_ capacity as 213 cm^3^ STP cm^−3^ outperforming its structural analogue PCN‐14 (197 cm^3^ STP cm^−3^) at 35 bar, 298 K as shown in **Figure** **5**a. This study showed that computational screening is an efficient way to discover promising MOFs and to direct experimental efforts to these materials. By sharing the CIFs and simulated CH_4_ uptake data of all hMOFs, Wilmer and co‐workers also played an important role in speeding up the design of MOFs for CH_4_ storage.

In the next year, Fernandez et al.^[^ ^151^ ^]^ screened 137 953 hMOFs for CH_4_ storage via GCMC simulations at 1, 35, and 100 bar, 298 K. They utilized QSPR analyses to get better insights into the complex relations between CH_4_ uptakes of MOFs and their structural and chemical properties. Results showed that MOFs with void fractions (0.6–0.8) and large pores (>10 Å) achieve high CH_4_ uptakes at 35 bar (>200 cm^3^ STP cm^−3^) and 100 bar (>250 cm^3^ STP cm^−3^). Snurr and co‐workers focused on 122 835 pcu‐hMOFs from Wilmer's database, and performed GCMC simulations at 5.8 and 65 bar, 298 K for calculating CH_4_ deliverable capacities.^[^ ^152^ ^]^ They found that hMOFs can achieve maximum CH_4_ deliverable capacity of 206 cm^3^ STP cm^−3^, outperforming well‐known synthesized MOFs such as HKUST‐1 (190 cm^3^ STP cm^−3^), NU‐125 (183 cm^3^ STP cm^−3^), and NU‐111 (179 cm^3^ STP cm^−3^). They also underlined that hMOFs with void fractions (<0.8), volumetric surface areas (<5000 m^2^ cm^−3^), and high heat of adsorption for CH_4_ (>15 kJ mol^−1^) are promising for storage and delivery applications.

In 2015, Wu and co‐workers filtered Wilmer's database and identified a total of top 1200 hMOFs considering i) CH_4_ adsorption per material weight and ii) CH_4_ adsorption per material volume at 35 bar, 298 K together with top materials based on iii) void fraction and iv) surface area.^[^ ^153^ ^]^ GCMC simulations were subsequently performed for these 1000 hMOFs at 5 bar, 358 K, and 170 bar, 298 K to compute their deliverable capacities. They found that when operating conditions were set to 298 K and 170 bar for compression and 358 K and 5 bar for release, top‐ranked MOFs can exceed the 90% of ARPA‐E target, showing the high potential of MOFs for CH_4_ storage at different operating conditions.

Simon and co‐workers collected over 650 000 materials from the libraries of CoRE MOF and hypothetical databases of zeolites, porous polymer networks (PPNs), MOFs, and ZIFs to examine whether a material meets the ARPA‐E target at the defined conditions.^[^ ^154^ ^]^ They performed GCMC simulations at 65 and 5.8 bar, 298 K to compute CH_4_ deliverable capacities. Figure 5b shows that the CH_4_ deliverable capacities of MOFs are higher than zeolites and comparable to some of the PPN materials, but still, these classes of materials currently being investigated are unlikely to meet the ARPA‐E target for CH_4_ storage at ANG conditions because this target was set based on economic reasons for competition with compressed natural gas rather than materials’ thermodynamic properties. In addition to ANG conditions, the potential of LNG‐ANG operation conditions (159 K, 6 bar for adsorption and 298 K, 5 bar for desorption) was studied to achieve the ARPA‐E target for onboard CH_4_ storage and delivery. In a recent study,^[^ ^156^ ^]^ GCMC simulations for 5446 CoRE MOFs without OMS were performed at liquefied natural gas (LNG)‐ANG conditions to identify the best MOF adsorbents. Among 193 MOFs exceeding the ARPA‐E target, DUT‐23 was synthesized and tested. It was shown that DUT‐23 exhibits higher deliverable capacities (≈373 cm^3^ STP cm^−3^) than other MOFs under LNG‐ANG conditions and high stability during cyclic LNG‐ANG operation.

Since CH_4_ storage is one of the applications that gained momentum after the establishment of MOF databases, this research area has also become one of the first into which ML methods have been integrated. In 2017, Pardakthi et al.^[^ ^155^ ^]^ conducted the first HTCS‐based ML study focusing on CH_4_ storage in MOFs. They used Wilmer's database and GCMC simulation results at 35 bar, 298 K to construct different ML models predicting the CH_4_ capacities of MOFs. As shown in Figure 5c, ML models trained by using structural and chemical features of MOFs give the highest prediction power, providing a robust platform for identifying the best MOF materials for CH_4_ storage. Using the same dataset, Wu et al.^[^ ^157^ ^]^ trained different ML models to predict the CH_4_ storage capacities of hMOFs based on Henry's constant of CH_4_ for 130 398 structures and showed that the density and gravimetric surface area are the most important features to predict CH_4_ uptakes.

The studies that we discussed so far focused on predicting CH_4_ uptakes of MOFs at a given pressure. In a recent work, Bae et al.^[^ ^158^ ^]^ developed an MC‐ML strategy to accurately predict the heat of adsorption for CH_4_ based on the data acquired from molecular simulations of 4951 CoRE MOFs. They then tested the ML model by using experimental input data of MOFs to estimate the heat of adsorption for CH_4_ and CH_4_ adsorption isotherms of MOFs at various temperatures. Results revealed that using these algorithms allows researchers to estimate the CH_4_ adsorption isotherms of MOFs at any temperature by only providing the information of pore volume and surface area and CH_4_ adsorption isotherms at 298 K of corresponding samples. This is a very good example to show how the combination of molecular simulation and ML algorithms aid in calculating such data very easily and accurately, which is hard to measure experimentally.

With the aim of accelerating the search for optimal MOFs for CH_4_ storage, Lee et al.^[^ ^159^ ^]^ integrated ML and an evolutionary algorithm to develop multispecies genetic algorithms and screen 247 trillion hMOFs. To assess the CH_4_ storage potential of such a massive MOF space, evolutionary algorithms that search the promising material using generation‐based evaluation control are useful since brute force algorithms to calculate the properties of each of the hMOFs are not practical. During the optimization of this multispecies algorithm to find the peak value of CH_4_ deliverable capacity, they performed GCMC simulations for 200 264 hMOFs at 65 bar, 298 K and 5.8 bar, 298 K, and trained ML models with the simulated data. Results showed that more than 200 hMOFs exceeded the CH_4_ working capacity over 200 cm^3^ STP cm^−3^ and 96 MOFs exceeded the highest reported CH_4_ working capacity of 208 cm^3^ STP cm^−3^ (MOF‐519).^[^ ^160^ ^]^ The analysis for high‐performing MOFs showed that tetrahedral indium cluster, (metallo)porphyrins, and complexes with double‐bonds and triple‐bonds frequently occur among the high‐performing MOFs. This study demonstrated that a combination of recent computational approaches with molecular simulations can significantly improve the conventional way of doing HTCS studies in examining MOFs’ performance limits for CH_4_ storage applications to meet industrial targets.

### 4.3 H_2_ Storage

The goal of utilizing H_2_ as an efficient and environmentally friendly energy carrier for transportation is a challenging yet crucial endeavor, with significant research currently in progress. Thanks to its lower volumetric energy density compared to traditional fuels (8 MJ L^−1^ for liquid H_2_ vs 32 MJ L^−1^ for gasoline),^[^ ^161^ ^]^ a substantial amount of H_2_ must be stored in vehicles to match the driving range of existing technologies. The DOE target has established benchmarks for the H_2_ storage capacity in terms of gravimetric and volumetric units, and it is expected to achieve a gravimetric H_2_ capacity of 6.5 wt% and a volumetric capacity of 50 g H_2_ L^−1^ to be competitive with traditional technologies, with a goal set for 2025 to reach 5.5 wt% and 40 g H_2_ L^−1^.^[^ ^162^ ^]^

**Figure 6:**
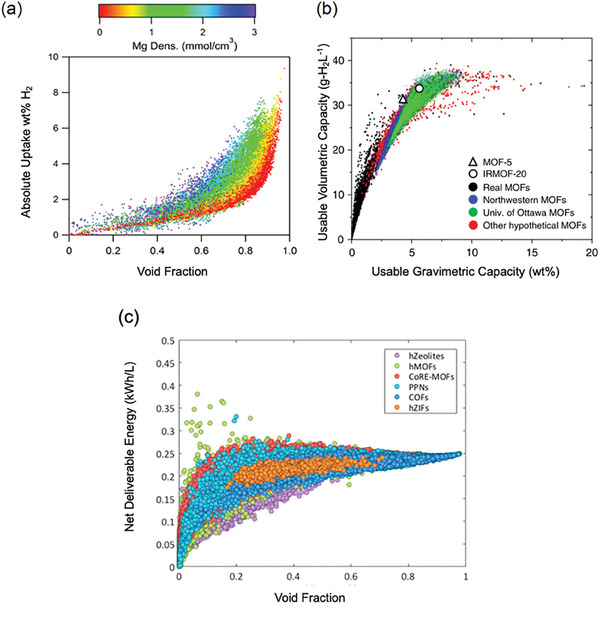
a) Relationship between void fraction and H_2_ uptake for 18 383 materials computed through GCMC simulations at 243 K, 100 bar. Reproduced with permission.^[^ ^164^ ^]^ Copyright 2014, American Chemical Society. b) Screening of 43 777 MOFs for H_2_ storage between 100 and 5 bar, at 77 K and comparison of well‐known MOFs (MOF‐5 and IRMOF‐20). Reproduced under the terms of the Creative Commons Attribution (CC‐BY) license.^[^ ^166^ ^]^ Copyright 2019, Springer Nature. c) Net deliverable energy at room temperature and cyclic process between 100 and 1 bar using the data acquired from GCMC simulations against the void fraction of structures. Reproduced under the terms of the Creative Commons Attribution (CC‐BY) license.^[^ ^167^ ^]^ Copyright 2017, American Chemical Society.

Before molecular simulations were utilized to screen MOFs, Goldsmith et al.^[^ ^163^ ^]^ used the Chahine rule, which is based on the linear relation between gravimetric H_2_ uptake and accessible surface area of porous materials, and calculated gravimetric and volumetric H_2_ uptakes at 35 bar, 77 K for 22 700 synthesized MOFs compiled from the CSD database in 2013. This first large‐scale study on the H_2_ storage capacities of MOFs showed that 76 MOFs can achieve higher H_2_ storage performance than the DOE target with volumetric uptakes between 40 and ≈70 g L^−1^ and gravimetric uptakes between 5.5 and 19.7 wt% at 77 K, 35 bar. Colón et al.^[^ ^164^ ^]^ conducted GCMC simulations for hypothetically constructed 18 383 magnesium‐oxide functionalized MOFs and showed that MOFs can achieve H_2_ deliverable capacities up to 9.12 wt% at 243 K for the pressures of 2 and 100 bar. The structure–performance relationship also showed that H_2_ deliverable capacities were maximized when the MOFs’ void fractions were ≈0.75 and Mg densities in MOFs were ≈2.5 mmol cm^−3^ as shown in **Figure** **6**a.

Chen et al.^[^ ^165^ ^]^ constructed 2800 hMOFs using the ToBaCCo code and performed GCMC simulations to compute their H_2_ deliverable capacities at 77 K, 100 bar, and 160 K, 5 bar. Among the studied MOFs, NU‐1501‐Al was selected based on the optimum structural properties, including gravimetric and volumetric surface areas, largest pore diameter, and void fraction. NU‐1501‐Al was subsequently synthesized and tested for H_2_ storage, and its deliverable capacity was reported as 46.2 g L^−1^. Bobbitt et al.^[^ ^122a^ ^]^ utilized GCMC simulations to compute H_2_ uptakes of 137 953 hMOFs constructed by Wilmer et al.^[^ ^34^ ^]^ at 2 and 100 bar, 77 K. They showed that MOFs achieving higher H_2_ deliverable capacities than the DOE target tend to have surface areas of ≈2000 m^2^ cm^−3^, void fractions of ≈0.9, and the heat of adsorption in between 2 and 4 kJ mol^−1^. Siegel and co‐workers filtered 43 777 synthesized and hypothetical MOFs out of 493 458 structures by using the Chahine rule and computed their H_2_ uptakes by GCMC simulations at 5 and 100 bar, 77 K, as illustrated in Figure 6b.^[^ ^166^ ^]^ MOFs with high simulated uptakes, such as PCN‐610, SNU‐70, ZELROZ, and UMCM‐9 were synthesized and shown to exhibit higher H_2_ uptakes than MOF‐5 and IRMOF‐20 (white triangle and circle in Figure 6b, respectively), which is a key discovery and example of how molecular simulations can direct the experimental efforts to find the best materials.

Thornton et al.^[^ ^167^ ^]^ conducted the first ML study for H_2_ storage in MOFs for more than 850 000 materials consisting of MOFs, COFs, PPNs, and zeolites at pressures of 1 and 100 bar, 77 K to train a neural network ML algorithm. As illustrated in Figure 6c, hMOF adsorbents with very low void fractions (≈0.1) offer the highest net deliverable energies (≈0.4 kW h L^−1^) acquired from the results of GCMC simulations for room temperature H_2_ storage, which is significantly less than the net energy delivered by high compression systems, 1.2 kW h L^−1^. A neural network‐based ML model was then developed to predict the H_2_ deliverable capacities for the entire dataset using the GCMC simulation results of 3000 materials as inputs for cryogenic storage conditions at 77 K. Results showed that the predicted H_2_ deliverable capacities reach up to 40 g H_2_ L^−1^ with net deliverable energy of 1.3 kW h L^−1^, surpassing the proposed value for compressed H_2_ in an empty tank at 700 bar.

Ahmed and Siegel developed an ML model to predict the H_2_ deliverable capacities of 918 734 experimentally synthesized MOFs and hMOFs compiled from 19 databases using the H_2_ uptake data of 98 695 MOFs acquired from GCMC simulations under pressure swing and temperature swing conditions.^[^ ^168^ ^]^ Results showed that the top‐performing structures are mostly hMOFs exhibiting low densities (<0.31 g cm^−3^) along with high surface areas (>5300 m^2^ g^−1^), void fractions (0.90), and pore volumes (>3.3 cm^3^ g^−1^). Bucior et al.^[^ ^122b^ ^]^ integrated GCMC simulations with ML algorithms to evaluate 137 953 hMOFs and 54 776 synthesized MOFs for H_2_ storage at 2 and 100 bar, 77 K. GCMC simulations were first performed to compute H_2_ uptakes for 137 953 hMOFs. ML models were subsequently developed using 1D energy histograms as inputs, which were acquired from the potential energy landscape that represents a 3D map of H_2_–MOF interaction energies. Results showed that 51 top‐performing MOFs have high H_2_ deliverable capacities (>45 g L^−1^). MFU‐4*l* was selected and synthesized among these MOFs, and its H_2_ deliverable capacity was measured as 47 g L^−1^ when storage and delivery steps were performed at 100 bar, 77 K and at 5 bar, 160 K, respectively. These results demonstrated that MFU‐4*l* is among the top‐performing MOFs reported in the literature, such as Cu‐BTC (46 g L^−1^), NOTT‐112 (41 g L^−1^), and NU‐125 (49 g L^−1^).^[^ ^169^ ^]^

### 4.4 Water Harvesting

Atmospheric water is a major natural source as a solution to water scarcity. It is estimated that 10% of all freshwater sources exist in the air as vapor and moisture droplets. This corresponds to ≈13 000 trillion liters of fresh water, which could significantly contribute to solve the global water crisis.^[^ ^170^ ^]^ To harness this vast resource, there is an urgent need to develop new water harvester materials and MOFs have emerged as unique candidates with a high capability of trapping water even at low relative humidity conditions.

In the context of water adsorption in MOFs, hydrolytic stability is one of the key factors for developing MOFs for water harvesting. While MOF‐5 and Cu‐BTC have been widely studied for gas storage as we discussed above, their usage in water harvesting is limited due to their moisture sensitivity and degradation in the presence of water.^[^ ^171^ ^]^ The first studies for water adsorption started with the MOFs that were examined for CO_2_ separation under humid conditions.^[^ ^22^, ^172^ ^]^ MOFs were extensively tested for water harvesting for the first time in 2014 by Yaghi and co‐workers. MOF‐801 and MOF‐303 were shown as the current state‐of‐the‐art adsorbents as they can deliver ≈0.3 g of fresh water per gram of the MOF at arid conditions (20% relative humidity).^[^ ^173^ ^]^

The number of computational studies in this field is limited because molecular simulations of water adsorption are highly challenging for several reasons.^[^ ^174^ ^]^ i) In molecular simulations, the formation of hydrogen bonds between polar water molecules and MOF atoms can complicate energy calculations. ii) The water–MOF system may not easily reach equilibrium and stay stuck in a local free energy minimum due to the formation of adsorbed water (guest) clusters. iii) It is not straightforward to choose a water model that fully captures every important aspect of bulk water. iv) The lack of reliable experimental data on water adsorption affects the model validation. The complexity of modeling water adsorption in MOFs can increase if MOFs have special properties such as defects, OMSs, and structural flexibility, which need a special force field to define the unique adsorbent–water interactions. Therefore, early molecular simulations focused on only a few MOFs and to accurately represent the bulk water properties by developing force field parameters.^[^ ^175^ ^]^ In the last two years, there has been a rise in the number of molecular simulation studies to examine MOFs for water harvesting. For example, Hanikel et al.^[^ ^176^ ^]^ combined Monte Carlo simulations and DFT calculations to show that when the linker of MOF‐303 was changed, water uptake was boosted by 50% under 30% relative humidity, showing the impact of the linker on the water adsorption properties of MOFs.

The first HTCS study for water harvesting with MOFs was recently reported by performing GCMC simulations for 6013 MOFs to calculate their H_2_O selectivities from (O_2_ + N_2_) at infinite dilution, 298 K.^[^ ^177^ ^]^ Using the Henry's constant of H_2_O, O_2_, and N_2_, ML models were trained to predict H_2_O selectivity of MOFs and materials with high selectivity were found to have low porosity (0.2–0.6), narrow pore size with ≈0.4 nm and low surface area (<1423 m^2^ cm^−3^). Based on the computed H_2_O selectivities, 10 superhydrophilic CoRE MOFs were identified.

**Figure 7:**
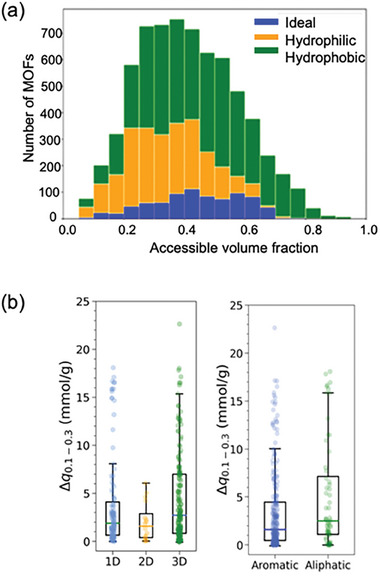
a) Relationship between the MOFs’ type and their geometric features, including accessible volume fraction. Reproduced with permission.^[^ ^178^ ^]^ Copyright 2024, American Chemical Society. b) Box plots of pore dimensionality and linker chemistry versus working capacity at low relative humidity (adsorption at 30% and desorption at 10% relative humidity) of collected MOFs. Reproduced with permission.^[^ ^179^ ^]^ Copyright 2023, American Chemical Society.

For modeling water adsorption in MOFs, different simulation methods other than GCMC were also used. Recently, Wang et al.^[^ ^178^ ^]^ performed the flat‐histogram Monte Carlo (MC) simulations for CoRE MOF database to classify the structures in terms of their hydrophilicity and to obtain their maximum H_2_O deliverable capacities. This simulation method uses a constant number of molecules (*N*), volume (*V*), and temperature (*T*) by computing the probability of acceptance ratio of a ghost molecule in the simulation box. The main idea is to sample the gas adsorption isotherms of MOFs only for a few selected number of adsorbed H_2_O molecules to determine their range of H_2_O loadings at a given pressure conditions. Results showed that 802 MOFs are “not too hydrophilic or too hydrophobic but potentially ideal” water harvesters in the CoRE MOF database. In addition, large‐pored MOFs, such as MOFs with higher accessible volume fractions as illustrated in **Figure** **7**a, tend to be hydrophobic, and vice versa for the more confined structures. Based on the maximum deliverable capacity of MOFs, 50% of the ideal MOFs potentially outperform current state‐of‐the‐art MOF adsorbents, such as MOF‐303 and MOF‐801 at 20% relative humidity, 298 K. To the best of our knowledge, there is no study on water harvesting that screens any MOF database other than CoRE MOFs, clearly showing that utilizing MOFs for water harvesting is still a substantially unexplored field.

Since simulated water adsorption data is limited, ML models predicting H_2_O adsorption properties such as Henry's constant of water, working capacities at different relative humidity regions, and maximum H_2_O uptakes have been trained with experimental data. For instance, in 2023, Zhang et al.^[^ ^179^ ^]^ collected 344 experimentally reported H_2_O adsorption isotherms in 285 MOFs from the literature and used them to develop an ML model for predicting H_2_O adsorption properties of 8059 CoRE MOFs. Among these MOFs, 149 MOFs were identified to have H_2_O uptakes ≥35 mol kg^−1^, 39 MOFs with working capacities at low humidity ≥10 mol kg^−1^, and 139 MOFs with working capacities at high humidity ≥8.7 mol kg^−1^. According to the structure–property relationship analysis given in Figure 7b and 3D MOFs or MOFs including aliphatic linkers exhibit higher working capacity at low relative humidity region (10–30%) compared to 2D MOFs or MOFs having aromatic linkers. Among the promising 39 MOFs, 36 MOFs with high working capacities at low relative humidity were reported as potentially water stable based on their computed Henry's constant values. This study is an important example of how experimental data accumulated in the literature can be combined with ML techniques to direct the design of MOFs for water harvesting.

In assessing the suitability of MOFs for various applications, different structural characteristics are preferred for different purposes. We conclude that MOFs with OMSs, functional groups, narrow pores, and less porous structures are most suitable for CO_2_ separation applications.^[^ ^66^, ^132^ ^]^ For storage applications of CO_2_, CH_4_, and H_2_, MOFs with high void fractions, large surface areas, and substantial pore volumes are ideal.^[^ ^152^, ^168^ ^]^ Conversely, for water harvesting applications, MOFs with narrow pores, low void fractions, and smaller surface areas are preferable.^[^ ^177^ ^]^

### 4.5 MOF‐Based Materials and COFs

In this review, we specifically focused on molecular simulation studies, which have significantly accelerated the design and development of MOFs for gas adsorption applications. Although they have not reached as many numbers and diversity as MOFs, COFs also have been studied in HTCS studies for CO_2_ capture,^[^ ^31^, ^32^, ^180^ ^]^ CH_4_ storage,^[^ ^32^, ^66^, ^181^ ^]^ and H_2_ storage.^[^ ^182^ ^]^ These molecular simulation studies have shown that COFs and hCOFs are promising materials as good as MOFs, demonstrating superior performance over traditional adsorbents like activated carbons and zeolites in terms of CO_2_ adsorption selectivities and working capacities for CO_2_/N_2_ and CO_2_/H_2_ separations,^[^ ^31^, ^32^, ^181^ ^]^ along with natural gas purification.^[^ ^181c,e^ ^]^ For example, Smit and co‐workers computed CO_2_/N_2_ mixture adsorption data of 69 840 hCOFs at PTSA condition by using GCMC simulations.^[^ ^32a^ ^]^ Results revealed that 72 hCOFs outperform MgMOF‐74 by achieving higher working capacities (>0.05 kg CO_2_ kg^−1^ adsorbent). Aksu et al.^[^ ^180d^ ^]^ screened the same database for CO_2_/H_2_ separation by utilizing GCMC simulations at 0.1, 1, and 10 bar, 298 K, mimicking PSA and VSA processes. They identified top‐performing hCOFs achieving very high CO_2_/H_2_ selectivities (up to 954), outperforming MOFs and COFs. COFs were also shown to achieve remarkably high CH_4_ deliverable capacities under standard conditions, exceeding the previously reported highest values for MOFs based on both experimental and simulation studies.

Smit and co‐workers utilized GCMC simulations to screen 69 840 hCOFs and computed their CH_4_ deliverable capacities in between 5.8 and 65 bar, 298 K.^[^ ^66h^ ^]^ Results showed that 304 hCOFs achieve very high CH_4_ deliverable capacities (>190 m^3^ STP m^−3^) outperforming experimentally synthesized COFs. Van Speybroeck and co‐workers computed CH_4_ deliverable capacities of their own ReDD‐COFFEE database comprising 268 697 hCOFs by utilizing GCMC simulations at 5.8 and 65 bar, 298 K.^[^ ^32b^ ^]^ Results revealed that these hCOFs can achieve CH_4_ similar deliverable capacities compared to top‐performing well‐known MOFs, such as MOF‐5 (182 cm^3^ STP cm^−3^) and HKUST‐1 (183 cm^3^ STP cm^−3^). Recently, ML methodologies have also been useful for evaluating this large hCOF material space. Jiang and co‐workers integrated active learning ML algorithms with GCMC simulations to screen and discover COFs with high CH_4_ storage capacities for a total of ≈540 000 structures.^[^ ^181b^ ^]^ Results revealed that five GCOFs can achieve CH_4_ storage capacities up to 222.2 cm^3^ cm^−3^ in between 5.8 and 65 bar, 298 K, surpassing the world records of 208.0 cm^3^ cm^−3^ from experiment, and 217.9 cm^3^ cm^−3^ from simulations, respectively. Recently, the potential of COFs for H_2_ deliverable capacities became notable, achieving levels that surpass the DOEs’ target for efficient H_2_ delivery. Zhong et al.^[^ ^182^ ^]^ utilized GCMC simulations to compute H_2_ deliverable capacities of 449 CoRE COFs and a total of 6893 hCOFs refined from both 69 840 hCOFs and their own GCOF database (471 990 hCOFs) at 100 bar, 77 K and 5 bar, 160 K conditions. Results showed that COFs can achieve H_2_ deliverable capacities up to 56.1 g L^−1^, and 99.5% of hCOFs achieve the DOE 2025 target (50 g L^−1^) for H_2_ delivery. The huge number of COF materials (≈800 000) shows us that screening this material database by integrating both ML and experimental techniques is important, and we anticipate a continued increase in studies focusing on COFs, particularly in applications related to CO_2_ capture. These efforts are expected to lead the way in the synthesis of unique COFs, similar to what has been observed in MOF research.

Ionic liquids (ILs), which are molten salts at room temperature have been incorporated into MOFs and COFs to design IL/MOF and IL/COF composites with high gas separation performance.^[^ ^183^ ^]^ Molecular simulation studies of these composites are limited since computationally expensive minimization steps and DFT calculations are required to incorporate ILs into the pores of MOFs and to optimize the IL structures, respectively. Polat et al.^[^ ^184^ ^]^ conducted GCMC simulations for 1085 different types of 1‐n‐butyl‐3‐methylimidazolium tetrafluoroborate ([BMIM][BF_4_])‐incorporated MOFs for CO_2_/N_2_ separation. Results showed that the decrease in pore sizes and porosities of MOFs upon IL incorporation improved the CO_2_/N_2_ selectivities. Zhong and co‐workers performed configurational‐bias Monte Carlo (CBMC) simulations to generate a total of 550 357 composites, which included 1,3‐dimethylimidazolium tetrafluoroborate, ([MMIM][BF_4_]) and 1‐n‐butyl‐3‐methylimidazolium bis(trifluoromethylsulfonyl)‐amide ([BMIM][Tf_2_N]) incorporated hMOFs for CO_2_/CH_4_ separation.^[^ ^66k^ ^]^ Topologies and pore sizes of hMOFs were found to be crucial in determining composites with high CO_2_/CH_4_ selectivities.^[^ ^66k^ ^]^ ML algorithms were combined with molecular simulations to study 941 types of [BMIM][BF_4_]/MOF composites for CO_2_/N_2_ separation. ML models trained using the simulated data of [BMIM][BF_4_]/MOF composites accurately predicted CO_2_ and N_2_ uptakes of unseen composites and successfully directed the synthesis of a new [BMIM][BF_4_]/UiO‐66 composite for CO_2_/N_2_ separation.^[^ ^185^ ^]^ Yan et al.^[^ ^186^ ^]^ performed CBMC simulations to incorporate [MMIM][BF_4_] into 557 CoRE COFs. CO_2_ and N_2_ uptakes of [MMIM][BF_4_]/COF composites were subsequently computed by performing GCMC simulations and were used as input data for developing ML models. Results showed that promising IL/COF composites exhibited favorable CO_2_/N_2_ separation at an IL loading of 35 vol%. These studies emphasize the pivotal role of computational screening and ML in directing experimental efforts toward highly promising materials and facilitating the design of new IL/MOF and IL/COF composites with exceptional CO_2_ separation performance.

Amorphous MOFs and MOF glasses: Apart from crystalline MOFs, amorphous MOF gels and MOF glasses have recently gained interest thanks to ease of fabrication and improved processability.^[^ ^187^ ^]^ Amorphous MOF gels typically consist of a network of MOF nanoparticles dispersed within a gel matrix whereas MOF glasses are derived from crystalline MOFs through thermal or mechanical treatment. GCMC and MD simulations were used to simulate amorphous MOFs and glasses for CO_2_, CH_4_, and H_2_ adsorption.^[^ ^188^ ^]^ Zhou et al.^[^ ^188a^ ^]^ reported the first MOF glass formed from ZIF‐76, and studied its CO_2_, CH_4_, and H_2_ adsorption using GCMC simulations. Mohamed and Kim utilized MD simulations to model an amorphous MOF, and performed GCMC simulations to compute CH_4_ and H_2_ uptakes.^[^ ^188b^ ^]^ They showed that the amorphous MOF can achieve higher CH_4_ and H_2_ uptakes than its perfect crystal version. Thyagarajan and Sholl computed CO_2_/CH_4_ mixture adsorption data of amorphous ZIF‐4 at 10 bar, 298 K by GCMC simulations and revealed that ZIF‐4 can achieve CO_2_/CH_4_ selectivity of ≈7, outperforming amorphous carbons.^[^ ^188c^ ^]^ Significant challenges still exist for modeling complex and disordered structures of amorphous MOFs, such as the sensitivity of MD simulations to chosen parameters and the difficulty of accurately characterizing amorphous states.^[^ ^189^ ^]^

## 5 Outlook

We have witnessed the transformative evolution of the MOF field driven by the synergistic interplay between experiments, molecular simulations, and AI‐based techniques. Initially grounded in the experimental synthesis and characterization of materials, the exploration of MOFs rapidly expanded as molecular simulations offered deep insights into their structural, functional, and performance properties. Today with the integration of AI, molecular simulations become a very powerful tool for accelerating the design and discovery of MOFs for a wide range of applications, mainly for gas adsorption. We addressed the current opportunities and challenges in the field to accelerate the adsorption applications of this fascinating material family below.

Accuracy of molecular simulations: Several assumptions are made in the molecular simulations of MOFs to reduce the computational costs. In HTCS studies, MOFs are typically assumed as perfect, rigid crystal structures, whereas experimentally synthesized MOFs may exhibit defects and some can have structural flexibility.^[^ ^190^ ^]^ Considering framework flexibility even for a single MOF significantly increases the computational cost and when thousands of MOF structures are studied, computing all these intra and intermolecular interactions is infeasible.

Generic force fields are commonly employed in HTCS studies to describe the interactions between gas molecules and framework atoms. However, these force fields might fail to accurately capture specific interactions if MOFs possess unique properties such as open metal sites and/or special functional groups within the frameworks, which necessitate the development of specialized force fields for accurately describing the framework–guest interactions for this type of MOFs. The need to remove the ambiguity and inaccuracies resulting from the generic force fields has driven researchers to leverage advanced quantum chemical methods such as DFT in molecular simulations. For example, using potential energy surface derived from DFT calculations within GCMC simulations will be important to accurately describe adsorbent–adsorbate interactions. By providing accurate energy predictions and structural insights, DFT calculations empower the parameterization of force fields, elevating the accuracy of simulations.^[^ ^96^, ^191^ ^]^ As a result of the assumptions that we discussed above, molecular simulations assuming a “perfect world” may either overestimate or underestimate the gas adsorption properties of MOFs. Hence, considering the distinctive characteristics of MOFs is essential for the customization of molecular simulation techniques to suit specific requirements. It is important to note that despite decades of use, there remains a significant gap in systematically assessing the accuracy of generic force fields specifically tailored for gas adsorption in MOFs. While these force fields have been extensively applied in molecular simulations and provided valuable insights into MOFs’ behavior, their precision and reliability in capturing the nuances of adsorption phenomena are not well‐validated. Comparative studies are needed to evaluate how well these force fields predict adsorption isotherms, the host–adsorbate interactions, and overall material performance. Such assessments are crucial for refining the force field parameters and enhancing the predictive power of simulations for MOF‐related applications.

Reproducibility of molecular simulations: To maximize the benefits of molecular simulation data for the scientific community, reproducibility checks should be done. The first thing to prove in a molecular simulation study is that the system is converged and equilibrated, which might be critical for complex problems such as water adsorption in MOFs. Thus, it is necessary to provide enough data for the reproduction and reuse of the simulations for additional uses. The computational methods section in the published papers is expected to include, at the very least, information on the simulation parameters along with the input and output files and codes.

The integration of interactive platforms with molecular simulation projects can offer a transformation in the field. Automated Interactive Infrastructure and Database for Computational Science (AiiDa)^[^ ^192^ ^]^ is an exemplary platform that provides a scalable infrastructure to manage workflows automating molecular simulations and the data generated via these simulations. This platform enables the sequential organization of various computations associated with structural analyses, DFT calculations, GCMC, and MD simulations of MOFs. Additionally, it processes further calculations from these simulations’ outputs to assess the performance of MOFs in gas storage and separation. The system also automates the storage of all input and output data on platforms like Materials Cloud,^[^ ^193^ ^]^ enhancing the accessibility and reproducibility of the data. Materials Project^[^ ^194^ ^]^ is another example that provides open access to large databases of materials properties, and combined with Python Materials Genomics (Pymatgen), it is possible to automate the workflow of simulations and data management. For example, the QMOF database under this platform stores both DFT‐based calculations of MOFs’ bandgap energies and input codes. Given the rapidly expanding range of MOFs, utilizing such platforms is essential for ensuring data reproducibility, standardizing calculations, and enhancing efficiency.

Acceleration of molecular simulations is also critical for reproducibility. The focus on central processing unit (CPU)‐based computation is common in many simulation packages, such as RASPA. However, CPU‐based simulations face limitations in handling large or complex systems, performing long simulations, and achieving extensive parallelism. This makes them slower compared to graphics processing unit (GPU)‐based approaches that excel in parallel processing of repetitive tasks. The shift toward GPU integration demands significant modifications to software architecture and algorithms to enhance molecular simulation speeds. Allowing simulation data to be produced faster will directly impact the design and discovery of MOFs in various applications.

Data management and standardization: To standardize molecular simulation data, the findable, accessible, interoperable, and reusable (FAIR) principles play a critical role in data management.^[^ ^195^ ^]^ A dataset organized and shared in accordance with FAIR principles can prevent the repeated execution of similar or related molecular simulations, which would accelerate modeling of MOFs and enable more efficient use of computational resources. It would be very important for the upcoming studies that provide simulation data to follow the FAIR principles. In addition, the adoption of standardized data formats in line with FAIR principles is crucial to accelerate the creation of structured datasets. Recently, the introduction of the adsorption information file (AIF) format^[^ ^196^ ^]^ has emerged as an important step toward simplifying the standardization of experimental and simulated adsorption data for future studies. Reporting the adsorption data in AIF format instead of plotting the full adsorption isotherms of MOFs offers a significant advantage by preventing the potential loss of information during the use of digitization methods. The JavaScript Object Notation (JSON) format has been recently utilized to present data from simulation outcomes alongside the parameters employed in these simulations, including the number of cycles and the type of force field utilized.^[^ ^66p^ ^]^ A recent contribution to the field is the CRAFTED database,^[^ ^197^ ^]^ Charge‐dependent, Reproducible, Accessible, Forcefield‐dependent, and Temperature‐dependent Exploratory Database, which includes the simulated CO_2_ and N_2_ adsorption data of 690 MOFs. This study aims to examine how force field and partial charge selection impact gas adsorption, providing valuable insights for the design of MOFs.

To promote open science in the field of porous materials for future researchers, application programming interfaces (APIs) serve as useful tools for sharing standardized data, particularly for accessing, analyzing, and integrating simulation data of MOFs. APIs promote interoperability and facilitate the exchange of data between databases and user applications, enabling advanced computational studies, materials discovery, and the development of AI‐based models for various applications.^[^ ^33^, ^66^ ^]^ In addition, correct accumulation of data is important in this process. For example, several research groups computationally focused on the same application, such as CO_2_ capture, but studied different MOF databases. Collecting these gas adsorption properties of different MOF databases will help identifying the theoretical performance limits of the vast MOF material space and guide the design of new MOFs with better adsorption performances. There has been a recent attempt to bring together simulated gas adsorption data of MOFs collected from distinct databases.^[^ ^66^, ^154^ ^]^ Recently developed large language models, such as ChatGPT,^[^ ^44^, ^198^ ^]^ and text and data mining tools^[^ ^199^ ^]^ can expedite the discovery of next‐generation materials by analyzing vast amounts of textual and numerical data of MOFs, and extracting valuable structure–property relationships from the literature.

Another point is the reusability of MOF structures in the previously published simulation studies. For example, a hypothetical database was constructed with trillions of MOF structures^[^ ^159^ ^]^ but its usability is limited since the structures are not publicly available. A related issue is valid for synthesized MOFs deposited into the CSD: Recent studies showed that MOFs with the identical CSD refcodes reported in different databases may exhibit varying chemical compositions because of the differences in the structure curation processes of the databases, such as solvent removal and the addition of missing hydrogens.^[^ ^200^ ^]^ Consequently, discrepancies in simulated gas uptakes among structures with identical reference codes may occur, particularly at lower pressures, affecting the selection of promising materials for a target application.^[^ ^200b^ ^]^ All these challenges in the field can be considered as new research directions related to data standardization and management in the field of computer simulations of MOFs.

AI‐enhanced molecular simulations: There is great potential to expand efforts that combine AI techniques with ab initio data to develop accurate force fields, which are expected to produce molecular simulation results that closely match experimental data, especially for MOFs for which generic force fields are not always sufficient. With AI integration, machine learning potentials (MLPs), which are trained using the results of DFT calculations, can be used in simulations to define dispersive and electrostatic interactions at the quantum level.^[^ ^201^ ^]^ For example, quantum‐informed machine learning force fields (QMLFFs) were introduced for the chemical adsorption and diffusion of CO_2_ in Mg‐MOF‐74, offering a computational efficiency ≈1000 times higher than classical simulations while maintaining quantum‐level accuracy.^[^ ^202^ ^]^ Simulations based on QMLFFs can accurately predict the landscape of binding free energy and the coefficient of diffusion, closely aligning with experimental values. MLPs were also developed to examine CO_2_ adsorption in ZIF‐8,^[^ ^203^ ^]^ H_2_ adsorption in Al‐soc‐MOF,^[^ ^204^ ^]^ and Xe diffusion in UiO‐66.^[^ ^205^ ^]^

These advancements also enhance the effectiveness and efficiency of molecular simulations across computationally demanding tasks. The molecular simulation programs have a large user base because they are reliable, functional, and practical. However, they are written in programming languages such as C/C++ or FORTRAN, and this can make it hard for simulation packages to keep pace with the ever‐evolving scientific computing techniques. AI frameworks, predominantly developed in Python, offer the benefit of quicker iteration and the flexibility to experiment more freely.^[^ ^206^ ^]^ While MLPs are computationally more demanding than classical force fields, the introduction of AI frameworks such as Tensorflow^[^ ^207^ ^]^ or Pytorch^[^ ^208^ ^]^ allows for efficient parallelization with GPUs, allowing these methods to become ever more used in research. AI‐boosted simulations achieve high efficiency on a wide array of computational devices, including CPUs and GPUs, and specialized units like tensor and neural processing units (TPUs and NPUs), ensuring broad applicability and performance optimization across different platforms. For example, MindSPONGE, an AI‐enhanced simulation package developed based on the MindSpore framework (an open‐source deep learning platform), has the capability to run advanced MD simulations for free energy calculations by easily integrating ML‐learned force fields to increase the efficiency of simulations. To the best of our knowledge, such AI‐enhanced simulation methods have not been applied to MOFs yet; however, given their promising advantages, future work might investigate their use in the MOF field.

Harnessing experiments, simulations, and data science to revolutionize the MOF field: In the field of MOF research, integration of experiments, simulations, and data science represents a promising approach for advancing our understanding of these fascinating materials. The traditional approach is first using computationally demanding molecular simulations at the HTCS level and then narrowing down the large numbers of MOFs to a small list of promising materials for further testing in the laboratory. With the integration of data science, studies have primarily focused on training ML models based on the molecular simulation data of a representative number of MOFs and then using these models to make quick predictions for the new materials without the need for simulations. In this data‐driven approach, of course, the accuracy of the ML models strongly depends on the accuracy of the molecular simulations. Harnessing experimental and computational methodologies also presents challenges, such as differences in modeling structures and inconsistencies in synthesis, characterization, and testing processes. Addressing these challenges requires strategies, including the establishment of standardized experiment and simulation protocols and stronger collaboration between research groups. In future studies, active learning (AL) can also play a guiding role in optimizing molecular simulations by predicting which simulations need to be performed. Instead of randomly choosing which MOF to study next, these models can help choosing the next MOF to study, thereby preventing the waste of computational time and resources. Future research directions may involve advancing AL models to effectively integrate simulation and experimental data together as well as exploring emerging areas such as materials informatics and innovative computational methods.

While closing, we would like to highlight the importance and strong need for collaboration between experimental and computational studies. As we have reviewed, only a limited number of MOFs discovered through computational screening has been experimentally synthesized despite the huge efforts in this field. In several computational studies, many high‐performing materials were discovered, and several new materials were in silico designed, but the challenges such as the difficulties related to the synthesis of these MOFs in the laboratory, or the chemical and mechanical stability of the discovered MOFs might slow down the transition of new MOFs from computer to real life. To overcome these challenges, the development and implementation of increasingly sophisticated computational approaches are expected to be integrated with experiments. Thus, we see a great opportunity for enhancing the interdisciplinary collaboration between experimentalists, simulators, and data scientists, to realize the full potential of MOFs in adsorption applications and to create a better research integrity with the open‐access sharing and reproducibility of MOF data.

## Conflict of Interest

The authors declare no conflict of interest.
